# Inter-niche and inter-individual variation in gut microbial community assessment using stool, rectal swab, and mucosal samples

**DOI:** 10.1038/s41598-018-22408-4

**Published:** 2018-03-07

**Authors:** Roshonda B. Jones, Xiangzhu Zhu, Emili Moan, Harvey J. Murff, Reid M. Ness, Douglas L. Seidner, Shan Sun, Chang Yu, Qi Dai, Anthony A. Fodor, M. Andrea Azcarate-Peril, Martha J. Shrubsole

**Affiliations:** 1 Department of Bioinformatics and Genomics, University of North Carolina at Charlotte, Charlotte, NC USA; 2 Department of Medicine, Division of Epidemiology, Vanderbilt Epidemiology Center, Vanderbilt University School of Medicine, Vanderbilt-Ingram Cancer Center, Vanderbilt University Medical Center, Nashville, TN USA; 3 Department of Statistics, North Carolina State University, Raleigh, NC 27695 USA; 4 Department of Biostatistics, Vanderbilt University School of Medicine, Nashville, TN USA; 5 Department of Medicine, Division of Gastroenterology, Hepatology, and Nutrition, Vanderbilt University School of Medicine, Nashville, TN USA; 6 Department of Medicine, and Microbiome Core Facility, School of Medicine, University of North Carolina, Chapel Hill, NC USA

## Abstract

The purpose of this study is to evaluate similarities and differences in gut bacterial measurements and stability in the microbial communities of three different types of samples that could be used to assess different niches of the gut microbiome: rectal swab, stool, and normal rectal mucosa samples. In swab-stool comparisons, there were substantial taxa differences with some taxa varying largely by sample type (e.g. Thermaceae), inter-individual subject variation (e.g. Desulfovibrionaceae), or by both sample type and participant (e.g. Enterobacteriaceae). Comparing all three sample types with whole-genome metagenome shotgun sequencing, swab samples were much closer to stool samples than mucosa samples although all KEGG functional Level 1 and Level 2 pathways were significantly different across all sample types (e.g. transcription and environmental adaptation). However, the individual signature of participants was also observed and was largely stable between two time points. Thus, we found that while the distribution of some taxa was associated with these different sampling techniques, other taxa largely reflected individual differences in the microbial community that were insensitive to sampling technique. There is substantial variability in the assessment of the gut microbial community according to the type of sample.

## Introduction

With recent advances in next-generation sequencing, our understanding of the role of the microbiome in health has dramatically increased^1–6^. The human colorectum microbiome is responsible for a substantial number of physiological functions that have both localized and systemic effects on human health including immunity, nutrient metabolism, growth, and energy harvesting^3,7–9^. Compositional shifts in the diversity or relative distributions of members of the gut microbiota have begun to be linked to several diseases^1–6^.

In the colorectum, there are different niches in which the gut microbiota may reside or interact with the human mucosal microenvironment. In addition to bacterial communities in the lumen, the epithelium is also covered by a mucus layer in which bacterial communities have been found. The function and prevalence of the microbiota within the colorectum likely varies by these niches (i.e. luminal vs. adherent mucosa)^10–12^. For example, studies have found the colon has the steepest oxygen gradient in the body, with anoxia sharply increasing from the mucosa to the middle of lumen^13^. Thus, luminal microbes are more likely to be anaerobic than mucosal communities^12^. Anaerobic luminal bacteria may play a key role in fermentation and metabolism of luminal contents (e.g. nutrients or carcinogens)^13–15^ while mucosal bacteria may be involved with autoimmune functions. In addition to differences in oxygen gradients, previous studies have found adherent mucosal communities are less diverse than luminal bacteria although they share many of the same predominant species^10,11,13,15–17^.

Differences in microbial communities are not only driven by niche, but can also reflect differences between individual human hosts. There have been many studies, including the Human Microbiome Project (HMP), that have observed more between-person variation than within-person variation leading to the conclusion that adults have an average unique microbial signature that is largely stable over time^10,18–22^. Individual microbial signatures have also been demonstrated in longitudinal studies. Rajilić-Stojanović *et al*. collected stool samples up to 9 times from 5 individuals over a decade and found that although there were some changes in abundances with age, individual-specific patterns persisted^18^. The findings from their study also indicated a single spot stool sample was not able to capture the presence of all core colonizers.

Many studies of the gut microbiome rely on collection and characterization of stool samples. Although stool may be the most convenient sample, it may only capture information about luminal bacteria which are more transient compared to adherent bacteria. Unfortunately, collection of mucosal samples by biopsy is a highly invasive procedure with risks of perforation that make it unfeasible for large-scale studies. Rectal swabs may prove a simple and inexpensive collection method that may sample both mucosal and luminal communities. Previous comparisons between swab and mucosal samples have shown that swab samples may capture many of the same bacteria as mucosal biopsy samples but also may be different than stool samples^12,16,23^. However, these previous studies have been small, did not compare samples from the same individual, collected samples post-bowel cleansing and/or did not make these comparisons using whole-genome shotgun sequencing (WGS). In addition, no previous study has evaluated within-person variation or stability for rectal swabs despite swabs being a collection method that may be more feasibly collected in a clinical setting than either stool or mucosal samples. In this study, we compared the microbial composition of stool samples, rectal swabs, and histologically normal rectal mucosa using both 16S rRNA amplicon sequencing and whole genome shotgun (WGS) sequencing and also compare composition of samples collected at two time points.

## Methods

### Study Population

The participants in this study were selected from the Personalized Prevention of Colorectal Cancer Trial (PPCCT), an on-going, double-blind, placebo-controlled, randomized clinical trial of 12 weeks designed to test the interaction between *TRPM7* genotype and reduction of calcium/magnesium intake ratio by magnesium supplementation on colorectal carcinogenesis biomarkers. The study is registered at ClinicalTrials.gov (NCT01105169). The study was approved by the Vanderbilt Institutional Review Board. All study procedures were performed in accordance with relevant guidelines and regulations as approved by the Vanderbilt Institutional Review Board.

Eligibility for the parent trial included 40–85 years of age, in good health, ability to participate in a supplement intervention, personal history of colorectal polyps, known *TRPM7* rs8042919 genotype, and daily calcium intake between 700–2000 mg/day and a ratio of daily intake of calcium and magnesium greater than 2.6. Exclusion criteria included any personal history of cancer other than non-melanoma skin cancer, colon resection or colectomy, gastric bypass, organ transplantation, inflammatory bowel disease, chronic diarrhea, chronic renal diseases, hepatic cirrhosis, chronic ischemic heart disease, or Type I diabetes mellitus. Also excluded were individuals using medications that may potentially interact with magnesium, or who were breastfeeding or pregnant. Eligible participants were randomized to receive either placebo (microcrystalline cellulose) or personalized magnesium supplementation (magnesium glycinate) for twelve weeks. Participants, health care providers and investigators were blinded to treatment assignments.

Participants included in the analysis reported here were selected and assayed at two different time points from individuals with biospecimens who had completed the trial at the time of selection. Participants were excluded if they used oral or injected antibiotics in the past 12 months before the study or during the study period. Selection for this analysis was prioritized to include individuals with a history of colorectal adenoma and known recurrence status (50% with recurrence, 50% without recurrence), with the less common *TRPM7* GA/AA genotype (n = 18), or for whom immunohistochemistry findings were completed (n = 51). From these criteria, individuals were randomly selected such that 50% were from the placebo arm and 50% from the treatment arm. A total of 60 individuals were selected from 150 participants enrolled between 4/11/2011 and 12/11/2013. Characteristics of the study participants included in this analysis are described in Supplementary Table S1.

### Sample Collection

Longitudinal samples were collected from the same subject at an interval of approximately three months. Participants collected stool samples at home up to 3 days prior to their in-person clinic visits. Stool was passed in to a white plastic collection container (Fisherbrand^TM^, Fisher Scientific, 02544208) covering the bowl of the toilet. Wearing gloves, participants scooped three portions of the stool in to four empty sterile feces collection containers (Sarstedt Inc, NC0705093). Samples were immediately frozen in their home freezer. They were also provided with a Styrofoam cooler and ice pack to use to transport the sample to the visit site. Upon receipt, the samples were placed in −80 °C freezers until future analysis. When possible, all stool samples for a participant were collected at the same time of day across the study period to avoid potential variability due to circadian rhythms. The mean (standard deviation) days between the collection and clinic visit was 1 (1.5) day.

At the clinic visits, the study physician inserted a culturette swab through the anal canal, swabbed the distal rectal mucosa, and immediately placed the swab into the empty storage vial. Rectal mucosal biopsies were then obtained through an anoscope using standard mucosal biopsy forceps and these samples were placed into separate storage vials. All the samples were immediately frozen at −80 °C until use. No colon cleansing preparation was used. The mean (standard deviation) days between the first sample collection and the last sample collection was 86.4 (6.6) days.

### DNA isolation

DNA was isolated using the E.Z.N.A. Stool DNA kit (Omega Biotek Inc., Norcross, GA) following manufacturer’s instructions including the optional incubation at 95 °C to ensure optimal lysis of Gram positive bacteria. After isolation, DNA was resuspended in 10 mM Tris-HCl (pH 8), aliquoted, and stored at −80 °C long term (before and after processing) or −20 °C short term (<2 weeks processing).

### 16S rRNA amplicon sequencing

16S rRNA amplicon sequencing was conducted for 60 participants. 12.5 ng of total bacterial DNA was amplified using primers consisting of a locus-specific portion targeting the V1–V2 region of the bacterial 16S rRNA gene using previously described primers^24,25^ and overhang adapter sequences appended to the primer pair for compatibility with the specific Illumina index and sequencing adapters. The complete sequences of the primers were: F – 5′ TCGTCGGCAGCGTCAGATGTGTATAAGAGACAGAGAGTTTGATCCTGGCTCAG 3′ and R – 5′ GTCTCGTGGGCTCGGAGATGTGTATAAGAGACAGGCTGCCTCCCGTAGGAGT 3′.

Master mixes for the first round of PCR reactions contained 2× KAPA HIFI HotStart ReadyMix (KAPA Biosystems, Wilmington, MA). The thermal profile for the amplification of each sample had an initial denaturing step at 95 °C of 3 minutes, followed by a cycling of denaturing of 95 °C for 30 seconds, annealing at 55 °C for 30 seconds and a 30 second extension at 72 °C (25 cycles), a 5 minute extension at 72 °C and a final hold at 4 °C. Each 16S amplicon was purified using the AMPure XP reagent (Beckman Coulter, Indianapolis, IN) as recommended by the manufacturer. Next, each sample was amplified using a limited cycle PCR program, adding Illumina sequencing adapters and dual‐index barcodes (index 1(i7) and index 2(i5)) (Illumina, San Diego, CA) to the amplicon target. The thermal profile for sample amplification had an initial denaturing step at 95 °C of 3 minutes, followed by a cycling of denaturing of 95 °C for 30 seconds, annealing at 55 °C for 30 seconds and a 30 second extension at 72 °C (8 cycles), a 5 minute extension at 72 °C and a final hold at 4 °C. The final libraries were again purified using the AMPure XP reagent quantified and normalized prior to pooling. The DNA library pool was then denatured with NaOH, diluted with hybridization buffer and heat denatured before loading on the MiSeq reagent cartridge (Illumina) and on the MiSeq instrument (Illumina). Automated cluster generation and paired-end sequencing with dual reads were performed per the manufacturer’s instructions.

### Whole-genome shotgun metagenomics DNA sequencing

Whole-genome shotgun metagenomics (WGS) DNA sequencing was conducted for 50 participants including 100 stool samples, 28 rectal swabs, and 16 mucosa samples. For library preparation, 1 ng of intact genomic DNA was processed using the Nextera XT DNA Sample Preparation Kit (Illumina). The target DNA was simultaneously fragmented and tagged by the Nextera Enzyme Mix containing transposome that fragments the input DNA and adds the bridge PCR (bPCR)-compatible adaptors required for binding and clustering on the flowcell. Next, DNA was amplified using a limited-cycle PCR program adding index 1(i7) and index 2(i5) (Illumina) in unique combinations and sequences specific for cluster formation. The thermal profile for the amplification had an initial extension step at 72 °C for 3 minutes and initial denaturing step at 95 °C for 30 seconds, followed by 12 cycles of denaturing of 95 °C for 10 seconds, annealing at 55 °C for 30 seconds, a 30 second extension at 72 °C, and a final extension for 5 minutes at 72 °C. The library DNA then was purified using the Agencourt® AMPure® XP Reagent. Each sample was quantified and normalized prior to pooling. The DNA library pool was heat denatured before loading on the MiSeq reagent cartridge and on the MiSeq instrument. Automated cluster generation and paired-end sequencing with dual reads were performed per the manufacturer’s instructions.

### Microbial Classification

#### Preprocessing of 16S rRNA Gene Sequences

Raw Illumina base call output (BCL) obtained from the MiSeq were converted, but not demultiplexed, to paired-end fastq files using CASAVA^26^. The resulting paired-end fastq files were joined into single-end reads using fastq-join^27,28^. Quality filtering was applied to the output of fastq-join requiring that greater than 80% of the base pairs be specified with a quality score of at least 25 for a read to be retained. The quality filtered reads were then demultiplexed. Reads whose index sequence was not an exact match to the specified barcode were eliminated **(**Table 1**)**. Demultiplexed reads followed the QIIME^29^ split_libraries.py output convention and were suitable for subsequent analysis.

**Table 1:** 16S rRNA sequence reads from both stool and swab samples after various filtering steps.

Step	Number of Samples	Number of OTUs	Total Number of Sequence Reads	Mean Reads per sample ± SD (SE)	Minimum reads per sample	Maximum reads per sample
16S rRNA amplicon reads generated	240	—	36,832,742	153,469.76 ± 214,793.55 (13,864.86)	85	1,768,150
After clustering into closed-reference OTUs	240	8,375	36,826,591	153,444.13 ± 213,926.13	85	1,768,150
After filtering out OTUs in less than 20% of samples	240	1,849	32,222,426	134,260.11 ± 209,547.62	85	1,768,150

Demultiplexed 16S rRNA gene sequence reads were clustered into closed-reference Operational Taxonomic Units (OTUs) against the GreenGenes database^30^ using QIIME. For QIIME closed-reference picking, OTUs were picked with UCLUST at a similarity threshold of 0.97^31^. As a result, 8,375 OTUs were clustered with 8,373 classified at the phylum level, 4,052 classified at genus level and 0.23% reads remaining unclassified.

OTU count tables were then normalized as follows:

1 $${\mathrm{log}}_{10}\,(\frac{Bacteria\,count\,for\,sample\,\,i}{Number\,of\,sequences\,in\,sample\,i}\ast Average\,\#\,of\,sequences\,per\,sample+1)$$

Equation (1) minimizes differences in the impact of adding the pseudo-count of 1 to each sample. OTUs absent in more than 75% of the samples were discarded.

In order to ensure that our method of normalization or our use of closed-reference OTUs did not unduly bias our results, we performed and additional analysis (Supplementary Figures S1–S3), using QIIME open-reference picking and rarefication to 10,000 sequences. In our QIIME open-reference picking pipeline, OTUs were picked with the method uclust at a similarity threshold of 0.97. OTUs with cluster less than 5 sequences were discarded. As a result, 18,162 OTUs were clustered with 15,719 of these classified at phylum level and 6,378 classified at genus and 2.16% of the reads remaining unclassified. The OTU table was then rarefied to 10,000 reads per sample. Samples with less than 10,000 reads were discarded and OTUs absent in more than 75% of the samples were discarded. OTU counts and taxonomic assignments using rarefied samples counts were then log-10 normalized with a pseudo-count of 1.

WGS sequences were also assigned taxonomy using Kraken and the taxonomic calls were normalized (Equation (1))^32^.

### WGS Functional Classification

Paired end FASTQ files containing WGS sequences were converted to FASTA format. After filtering sequences mapping to the human genome forward WGS sequence reads were then run against the KEGG protein database^33,34^ using BLAST^35^. BLAST hits that had an e-value of 1 × 10^−3^ were kept. KEGG pathway abundances were calculated using HUMAnN^36^. KEGG gene families and pathways which were not present in at least 20% of the samples were removed **(**Table 2**)** and counts were normalized (Equation (1)).

**Table 2:** Whole-genome metagenome shotgun sequence reads from both stool and swab samples after various filtering steps.

Step	Number of Samples	Total Number of Sequence Reads	Mean Reads per sample ± SD (SE)	Minimum reads per sample	Maximum reads per sample
After removing reads mapping to the human genome	128	25,720,978	200,945.1 ± 104,515.8 (9,237.98)	342	521,089
After removing samples with low read counts	127	25,720,636	202,524.7 ± 103,384.5 (9173.9)	13,405	521,089
After assigning reads to gene families	127	22,285,207	175,474.1 ± 97,062.82 (8612.93)	13,863	455,173

### Multidimensional Scaling (MDS)

Multidimensional scaling (MDS) was performed on normalized counts data generated by RDP classifier or Kraken (microbial classification) and BLAST (functional classifications) using Bray-Curtis dissimilarity. The R package “vegan”^37^ was used to calculate the MDS axes.

### Statistical Analysis

Descriptive statistics of mean (standard deviation) for continuous variables and frequencies for categorical variables were derived for characteristics of the study participants. For the analysis of 16S rRNA amplicon and whole-genome metagenome sequencing data, the linear mixed effects model implemented the function “lme” from the R package “nlme” to evaluate the amount of variance due to stool vs. swab measures by controlling for time of sample collection. The p-values were generated from an ANOVA of the mixed linear models.

2 $$\begin{array}{c}{\rm{Diversity}}\,{\rm{Index}}\,{\rm{OR}}\,{\rm{MDS}}\,{\rm{axis}}\,{\rm{OR}}\,{\rm{taxon}}\,{\rm{OR}}\,{\rm{kegg}}\,{\rm{function}}\\ \quad \quad \quad \quad =\,{\rm{sampleType}}+{\rm{timePoint}}+(1|{\rm{participant}})\end{array}$$

In this model (Equation (2)), the sample type (stool or swab (or mucosa for WGS sequences)), and time point are fixed effects while participant (ID of participant) is a random effect. We also evaluated the treatment effect (magnesium vs placebo) using a model similar to Equation (2) with “treatment” as an additional independent variable. Because there was no overall treatment arm effect, treatment was removed from all final models. The analysis was conducted for the first 15 MDS axes, all taxa and all KEGG gene families/pathways. ANOVA was used on the mixed effects models to test the null hypothesis that sample type and time point did not contribute to the model. In this discovery analysis, the false discovery rate (FDR) was set at 10% to adjust for multiple comparisons^38^. To determine differences in the amount of aerobic versus anaerobic bacteria in swab and stool samples we used a chi-square test of independence. Bacteria were labeled anaerobic or aerobic according to Bergey’s Manual of Systematic Bacteriology.

### Code Availability

All R code and taxonomic tables used for this implementation is available in the GitHub repository and can be found here: https://github.com/rbarner/swabVsStool.

### Data availability

The sequence reads (16S rRNA amplicon and whole-genome shotgun reads) used is available on the NCBI Short Read Archive and can be accessed with the accession code https://www.ncbi.nlm.nih.gov/bioproject/PRJNA397450.

### Ethics approval

Written informed consent was obtained from all study participants, and the parent study and study protocol were approved by the Institutional Review Board at Vanderbilt University Medical Center.

### Availability of data and material

The datasets generated and/or analyzed during the current study are available in the Github repository, https://github.com/rbarner/swabVsStool.

## Results

### Comparison of Taxa from 16S rRNA Amplicon Sequencing between Stool and Swab Samples

**Figure 1:**
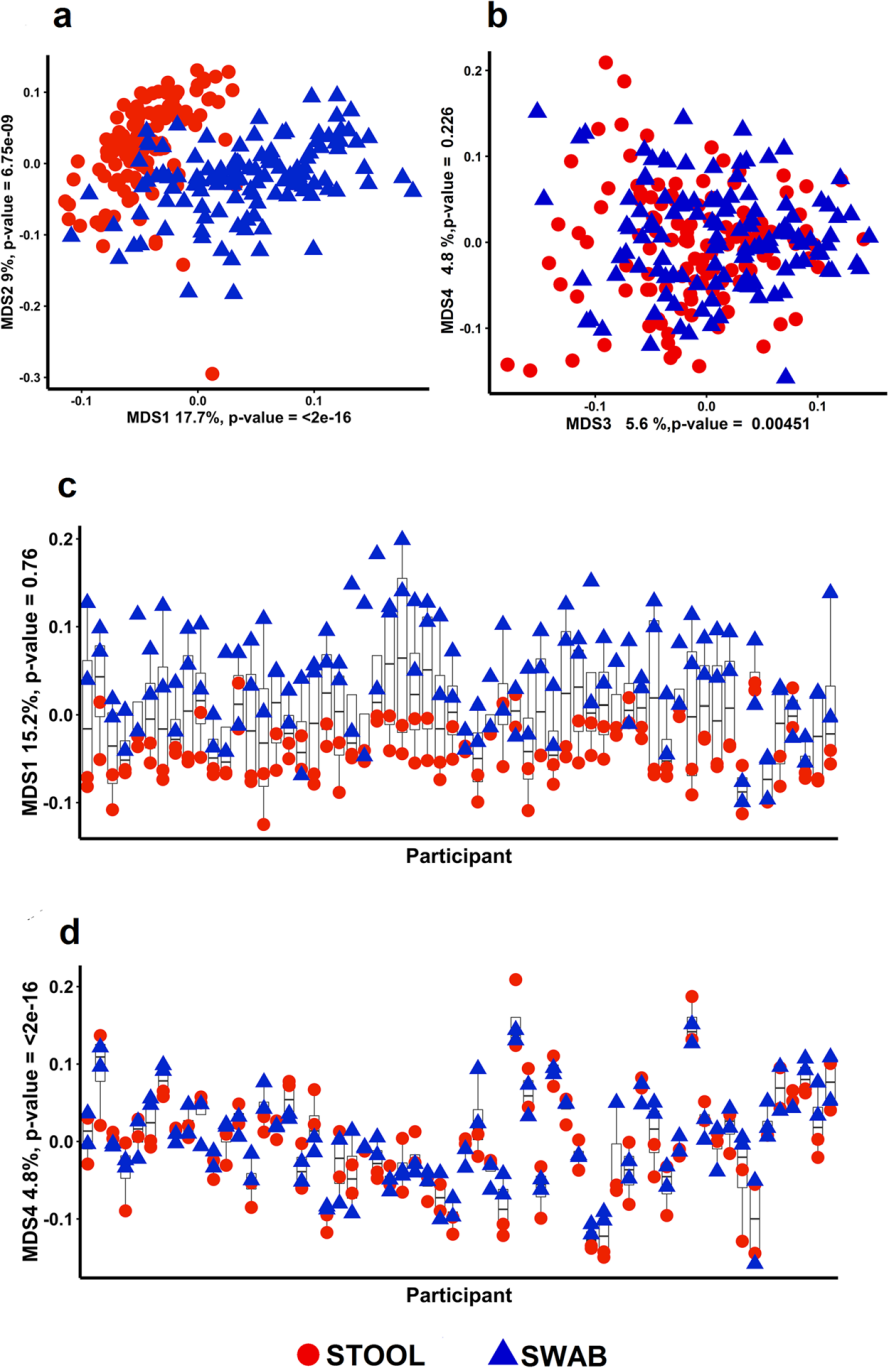
Multidimensional scaling (MDS) of closed-reference OTUs classified at the family level using 16S rRNA gene sequence reads. There are four samples (two each of stool and swab) from each of the 60 participants in our study colored by sample origin (red is stool and blue is swab). Repeated samples collected from individuals were collected with an average separation of 3 months. The distinct separation of colors shows that there is separation by sample type in MDS axis 1 and MDS axis 2 (**a**,**c**) but not in MDS axes 3 and 4 (**b**). However, MDS axis 4 shows strong clustering by participant (**d**).

**Figure 2:**
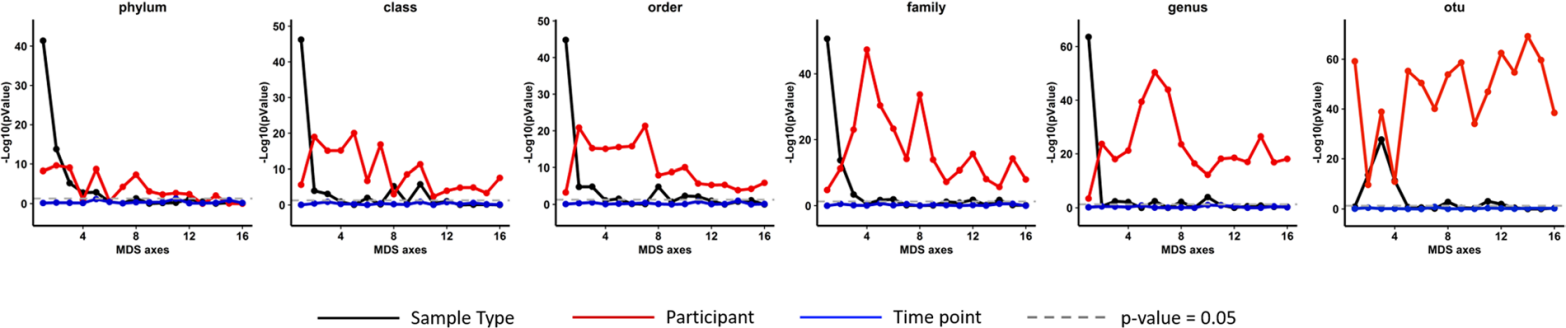
The first 15 MDS axes were regressed against sample type (swab or stool), participant ID and time point. The −log10 (p-value) for the null hypothesis that sample type, participant ID and time point have no impact on the MDS axes are all shown. While there are significant differences in the first MDS axis in stool vs swab samples, the MDS axes thereafter are significantly different between the participants. Taxonomic calls were based on QIIME closed-referenced OTU picking against GreenGenes database.

In a cohort recruited from an on-going NIH clinical study on the prevention of colorectal cancer (Supplementary Table S1), we collected stool and swab samples at two time points on average three months apart (see methods). We found substantial areas of similarity and differences between stool and swab samples based on the MDS ordination of 16S rRNA amplicon sequencing data (Fig. 1). The first two MDS axes (which accounted for almost 27% of the total variation of the microbial communities in the samples) showed nearly entirely distinct clusters between the stool (red symbols) and swab (blue symbols) (Fig. 1a). However, when comparing stool versus swab on two other MDS axes (Fig. 1b), there was a lack of distinct clusters. While the first MDS shows a difference between stool and swab that is consistent across all participants (Fig. 1c), the fourth MDS axis is much more influenced by each individual and appears to be less dependent on sample type (Fig. 1d). Also, to determine if there were significant differences in MDS clusters by sample origin, an analysis of variance was conducted using the function “adonis” in the R package “vegan”. This test showed that there is a significant difference between swab and stool in the dissimilarity matrix of the family level taxonomy counts (*p* = 8.01 × 10^−6^). To test whether the observed differences by sample type or participant were statistically significant, a linear mixed effects model was used with fixed effects for time point and sample type and a random effect for participant (Table 3). From the phylum to the OTU level, the first MDS axis was consistently associated with sample type (stool vs. swab). However, many other MDS axes had highly statistically significant effects for participant (Fig. 2) while showing no difference for sample type. This suggests that some taxa are highly specific to sample type within the colorectum (stool vs. swab) while other taxa are detectable with both sampling methods.

**Table 3:** Differences in MDS axes of closed-reference OTUs of 16S rRNA gene sequence reads classified to family level taxa due to sample source (stool vs. swab) and participant source.

Item	Stool vs. Swab					
	Stool samples (mean ± standard deviation)	Swab samples (mean ± standard deviation)	*p* value^a^	Participant *p* value^a^	R-squared Marginal^b^	R-squared Conditional^c^
**Analysis of MDS axes**						
Number of sequences per sample	139,959 ± 211,557	144,768 ± 191,410	0.848	0.099	0.005	0.097
Sun rarified Richness	10.44 ± 1.813	11.726 ± 2.348	3.00 × 10^−08^	4.80 × 10^−08^	0.087	0.386
Shannon diversity	1.83 ± 0.272	1.961 ± 0.311	1.55 × 10^−05^	1.36 × 10^−10^	0.049	0.418
Shannon evenness	0.514 ± 0.078	0.529 ± 0.08	0.095	2.74 × 10^−05^	0.009	0.255
MDS axis 1	−0.044 ± 0.03	0.045 ± 0.06	3.62 × 10^−37^	7.08 × 10^−05^	0.475	0.597
MDS axis 2	0.022 ± 0.07	−0.023 ± 0.05	1.20 × 10^−11^	3.92 × 10^−12^	0.127	0.496
MDS axis 3	−0.012 ± 0.06	0.012 ± 0.07	6.00 × 10^−05^	3.93 × 10^−23^	0.034	0.615
MDS axis 4	−0.005 ± 0.07	0.005 ± 0.06	0.0196	5.63 × 10^−47^	0.006	0.801
MDS axis 5	−0.008 ± 0.07	0.008 ± 0.06	0.001	1.58 × 10^−30^	0.026	0.688
MDS axis 6	0.009 ± 0.07	−0.009 ± 0.06	0.004	1.43 × 10^−23^	0.018	0.613
MDS axis 7	−0.002 ± 0.07	0.002 ± 0.06	0.557	1.40 × 10^−14^	0.005	0.472
MDS axis 8	0.001 ± 0.07	−0.001 ± 0.06	0.641	1.46 × 10^−33^	0	0.706
MDS axis 9	0.002 ± 0.07	−0.001 ± 0.07	0.809	2.05 × 10^−14^	0.002	0.483
MDS axis 10	−0.007 ± 0.07	0.006 ± 0.06	0.109	5.92 × 10^−08^	0.01	0.334
MDS axis 11	0.006 ± 0.07	−0.006 ± 0.06	0.109	2.35 × 10^−11^	0.008	0.416
MDS axis 12	−0.009 ± 0.07	0.009 ± 0.06	0.007	6.98 × 10^−16^	0.02	0.505
MDS axis 13	0.002 ± 0.06	−0.002 ± 0.07	0.557	8.64 × 10^−09^	0.001	0.35
MDS axis 14	0.009 ± 0.06	−0.008 ± 0.07	0.031	2.18 × 10^−06^	0.022	0.299
MDS axis 15	0.003 ± 0.07	−0.003 ± 0.06	0.438	1.34 × 10^−14^	0.008	0.475

^a^p-value derived from ANOVA of the mixed linear model. ^b^R-squared marginal represents the variation that is explained by the model without the mixed effect (participant) while ^c^the conditional R-squared represents the variation that is explained by the model including both fixed effects and mixed effects.

**Figure 3:**
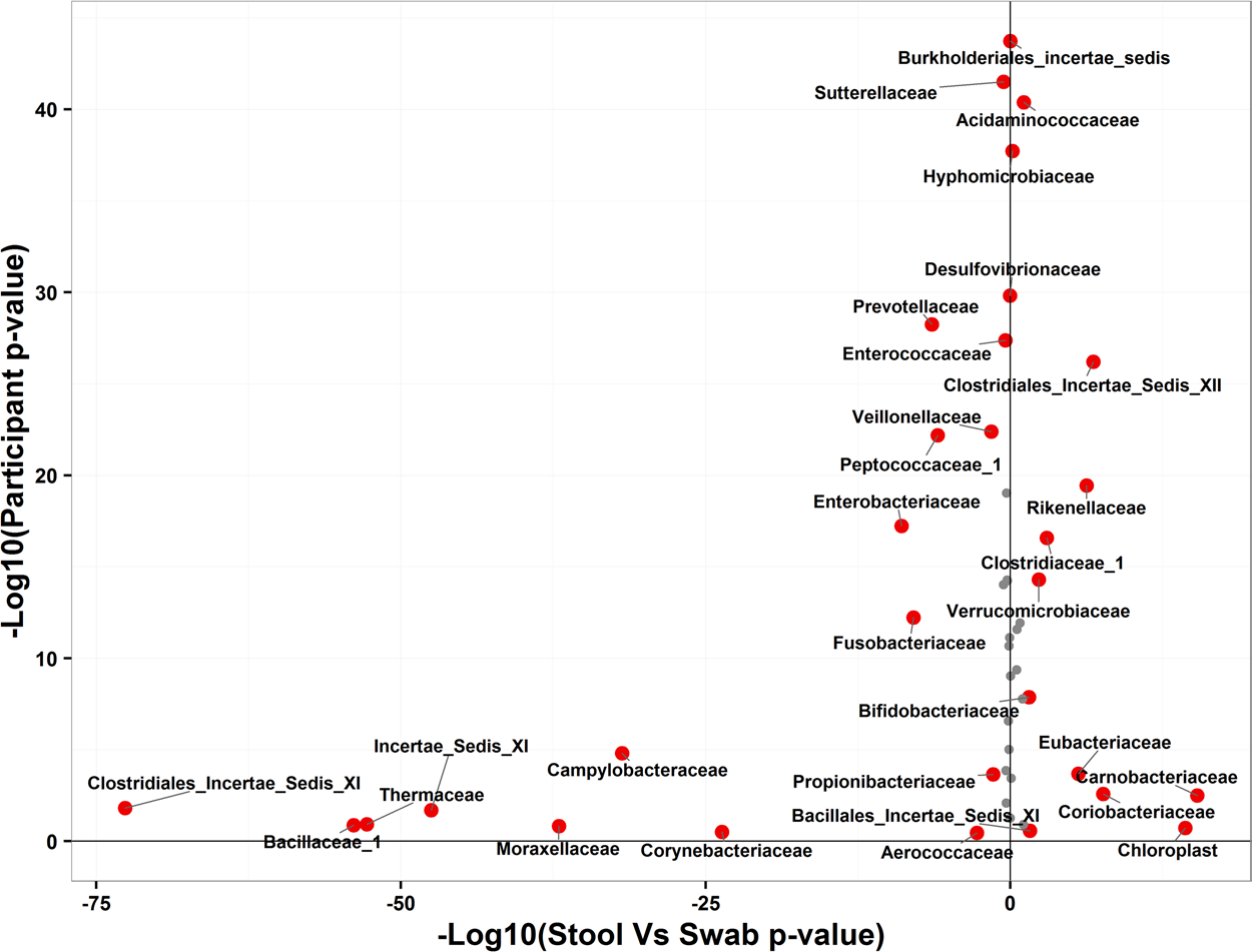
For each taxa at the family level present in at least 25% of samples, p-values for a null hypothesis of no difference by stool vs. swab vs. by participant. Red symbols are taxa that have a p-value that is significant at a 10% false discovery rate. Taxa higher in swab than stool have a negative x coordinate and taxa higher in stool than swab have a positive x-coordinate. Data was generated using closed-reference OTUs classified at the family level using 16S rRNA gene sequence reads.

In linear mixed effects models at the family level, 24 of 48 families were statistically significantly different between stool and swab samples while the variation in the logged abundance of 40 of 48 families showed significant variation due to the participant in which the sample originated (Table 4). There was substantial taxa by taxa variation (Fig. 3) in that there are families that vary by 1) sample type but not participant (e.g. Thermaceae), 2) participant but not sample type (e.g. Desulfovibrionaceae), or 3) both participant and sample type (e.g. Enterobacteriaceae) (Supplementary Figure S4). In addition, based on a chi-square test of independence, there was a statistically significant higher proportion (*p* = 0.02) of facultative aerobic genera that were significantly more abundant in swab samples than in stool samples (e.g. *Acinetobacter*, *Anoxybacillus*, and *Geobacillus*; Supplementary Table S4) consistent with a decreasing aerobic microbiota gradient from the mucosa to the lumen of the colorectum. This finding was reproduced using open-reference OTU data (*p* = 0.03). At species-level resolution (with WGS sequences) we observed that bacteria associated with colorectal tumors in previous studies^39^ (*Escherichia coli* and *Fusobacterium nucleatum*) were of higher relative abundance in swab than stool. There were also statistically significant differences in other bacteria according to the sample type (e.g. *Bifidobacterium longum*, *Bacteroides fragilis*) (Supplementary Table S5). Results using open-reference OTUs rarified to 10,000 reads show similar findings as data using relative abundance on closed-reference OTUs Supplementary Tables S2 and S3 and Supplementary Figures S1–S3 and S5. We also analyzed the differences in swab versus stool of each of the taxa using a non-parametric Wilcoxon test with taxon abundance as the dependent variable and sample source (swab versus stool) as the independent variable. These results were similar to those using the mixed models supporting the use of mixed models in this analysis (Supplemental Figure S6) suggesting that the assumptions of parametric models do not drive our results.

**Table 4:** Differences in closed-reference OTUs of 16S rRNA gene sequence reads classified to family level taxa due to sample source (stool vs. swab) and participant source.

Family Level Taxa^a^	Stool vs. Swab			Participant *p* value^c^	R-square Marginal^d^	R-squared Conditional^e^
	Log-Normalized Mean Abundance ± Standard Deviation					
	Stool	Swab	*p* value^b^			
Acidaminococcaceae	2.943 ± 1.1	2.728 ± 2.73	0.147	6.74 × 10^-40^	0.012	0.759
Actinomycetaceae	1.342 ± 0.69	1.327 ± 1.33	0.998	0.066	0	0.108
Aerococcaceae	0.169 ± 0.38	0.326 ± 0.33	0.004	0.341	0.027	0.078
Bacillaceae_1	1.866 ± 0.6	3.338 ± 3.34	3.18 × 10^-53^	0.148	0.482	0.526
BacillalesIncertae_Sedis_XI	0.702 ± 0.71	0.51 ± 0.51	0.055	0.278	0.016	0.076
Bacteroidaceae	4.564 ± 0.29	4.512 ± 4.51	0.275	2.88 × 10^-12^	0.011	0.436
Beijerinckiaceae	0.331 ± 0.51	0.304 ± 0.3	0.934	5.05 × 10^-04^	0.003	0.212
Bifidobacteriaceae	0.451 ± 0.6	0.317 ± 0.32	0.062	2.44 × 10^-08^	0.014	0.35
Burkholderiaceae	0.425 ± 0.62	0.499 ± 0.5	0.632	2.13 × 10^-04^	0.006	0.23
Burkholderialesincertae_sedis	0.697 ± 1.03	0.703 ± 0.7	0.998	8.92 × 10^-43^	0	0.779
Campylobacteraceae	0.331 ± 0.49	1.485 ± 1.49	1.10 × 10^-31^	2.47 × 10^-05^	0.304	0.482
Carnobacteriaceae	0.792 ± 0.68	0.32 ± 0.32	2.83 × 10^-15^	0.004	0.148	0.293
Chloroplast	0.751 ± 0.75	0.25 ± 0.25	2.30 × 10^-14^	0.197	0.138	0.201
Clostridiaceae_1	1.415 ± 1.15	1.033 ± 1.03	0.003	9.08 × 10^-17^	0.028	0.525
ClostridialesIncertae_SedisXI	0.884 ± 0.72	2.887 ± 2.89	1.13 × 10^-71^	0.019	0.611	0.665
ClostridialesIncertae_Sedis XII	1.403 ± 0.96	0.938 ± 0.94	5.60 × 10^-07^	3.71 × 10^-26^	0.076	0.664
ClostridialesIncertae_Sedis XIII	2.176 ± 0.7	2.076 ± 2.08	0.435	8.62 × 10^-10^	0.009	0.384
Comamonadaceae	0.195 ± 0.41	0.242 ± 0.24	0.648	0.011	0.003	0.154
Coriobacteriaceae	2.958 ± 0.56	2.652 ± 2.65	9.63 × 10^-08^	0.004	0.074	0.235
Corynebacteriaceae	0.105 ± 0.26	0.836 ± 0.84	1.52 × 10^-23^	0.320	0.226	0.269
Desulfovibrionaceae	1.91 ± 1.03	1.935 ± 1.94	0.998	1.43 × 10^-29^	0.001	0.672
Enterobacteriaceae	1.423 ± 1.16	2.129 ± 2.13	5.59 × 10^-09^	2.17 × 10^-17^	0.09	0.565
Enterococcaceae	0.296 ± 0.68	0.386 ± 0.39	0.562	2.87 × 10^-27^	0.006	0.649
Erysipelotrichaceae	3.189 ± 0.58	3.225 ± 3.23	0.863	4.53 × 10^-07^	0.009	0.315
Eubacteriaceae	0.859 ± 0.7	0.547 ± 0.55	7.28 × 10^-06^	0.000305	0.055	0.263
Flavobacteriaceae	0.362 ± 0.52	0.394 ± 0.39	0.921	4.53 × 10^-11^	0.001	0.406
Fusobacteriaceae	0.594 ± 0.97	1.199 ± 1.2	4.98 × 10^-08^	1.47 × 10^-12^	0.084	0.503
Hyphomicrobiaceae	0.96 ± 0.88	0.885 ± 0.89	0.800	2.24 × 10^-37^	0.004	0.74
Incertae_Sedis_XI	0.176 ± 0.37	1.618 ± 1.62	3.71 × 10^-47^	0.024	0.433	0.508
Lachnospiraceae	4.556 ± 0.19	4.512 ± 4.51	0.158	0.141	0.03	0.113
Lactobacillaceae	0.896 ± 0.84	0.947 ± 0.95	0.921	1.60 × 10^-05^	0.002	0.263
Microbacteriaceae	0.296 ± 0.58	0.353 ± 0.35	0.740	1.65 × 10^-14^	0.006	0.475
Micrococcaceae	0.436 ± 0.63	0.326 ± 0.33	0.177	2.95 × 10^-08^	0.009	0.346
Moraxellaceae	0.067 ± 0.26	0.986 ± 0.99	8.74 × 10^-37^	0.162	0.347	0.4
Pasteurellaceae	0.568 ± 0.81	0.55 ± 0.55	0.998	1.81 × 10^-09^	0.002	0.369
Peptococcaceae_1	0.416 ± 0.67	0.778 ± 0.78	3.33 × 10^-06^	3.15 × 10^-22^	0.06	0.614
Peptostreptococcaceae	1.924 ± 0.99	2.017 ± 2.02	0.717	1.62 × 10^-14^	0.003	0.479
Porphyromonadaceae	3.615 ± 0.67	3.683 ± 3.68	0.648	3.75 × 10^-19^	0.005	0.548
Prevotellaceae	2.633 ± 1	3.126 ± 3.13	1.27 × 10^-06^	4.60 × 10^28^	0.066	0.68
Propionibacteriaceae	0.172 ± 0.39	0.269 ± 0.27	0.077	0.000326	0.017	0.23
Rikenellaceae	3.356 ± 0.69	2.995 ± 3	1.77 × 10^-06^	1.60 × 10^-19^	0.052	0.576
Ruminococcaceae	4.368 ± 0.33	4.321 ± 4.32	0.435	6.04 × 10^-12^	0.009	0.434
Streptococcaceae	2.79 ± 0.77	2.825 ± 2.83	0.968	1.62 × 10^-11^	0.001	0.416
Sutterellaceae	2.569 ± 1.11	2.722 ± 2.72	0.435	7.57 × 10^-41^	0.01	0.767
Synergistaceae	0.544 ± 0.79	0.664 ± 0.66	0.435	2.57 × 10^-14^	0.007	0.473
Thermaceae	0.206 ± 0.4	1.581 ± 1.58	2.53 × 10^-52^	0.138	0.476	0.522
Veillonellaceae	2.225 ± 1.2	2.492 ± 2.49	0.061	2.15 × 10^-22^	0.017	0.599
Verrucomicrobiaceae	1.55 ± 1.22	1.202 ± 1.2	0.011	1.59 × 10^-14^	0.021	0.49

^a^Limited to families present in at least 25% of samples. ^b^Benjamini-Hochberg corrected p-value derived from ANOVA of mixed linear model. ^c^Benjamini-Hochberg corrected p-value derived from ANOVA of linear models with and without participant as a random effect. ^d^R-squared marginal represents the variation that is explained by the model without the mixed effect (participant) while ^e^the conditional R-squared represents the variation that is explained by the model including both fixed effects and mixed effects.

### Comparison of WGS Functional Classifications between Stool, Swab, and Mucosa Samples

**Figure 4:**
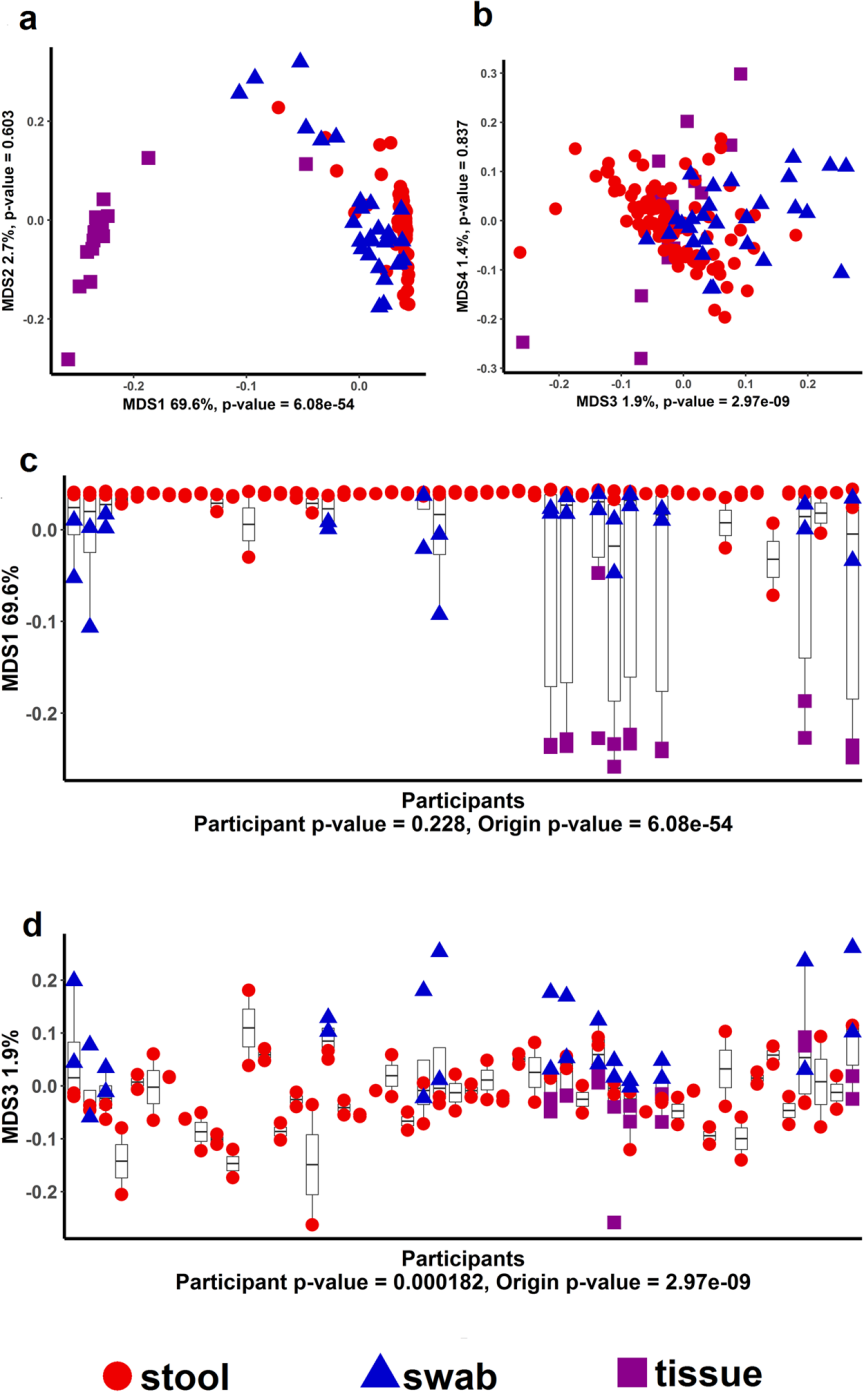
Plot of first two coordinates (**a**) and MDS3 vs MDS 4 (**b**) of an MDS ordination of the KEGG gene family abundance table for WGS sequence reads from swab samples (blue triangles), stool samples (red circles) and tissue samples (purple squares). The distinct separation of colors shows that there is separation by sample type in the first and third MDS axes. MDS axes plotted by participant ID show strong separation by sample type for MDS 1 (**c**) and by participant and sample type for MDS 3 (**d**).

To determine if sample type was distinct in microbial functions, we performed WGS on 50 participants including 100 stool samples, 28 rectal swabs, and 16 mucosa samples. We found pronounced differences in the functional pathways between the sample types based on MDS ordination for KEGG gene families using either all sample types including mucosa samples (Fig. 4) or using only stool and swab samples (Supplementary Figure S6), although stool and swab samples appear to be more closely related to each other than to mucosal biopsy samples. We also found that there was separation by sample type when we assigned taxonomy to WGS sequences (including mucosa samples (Supplementary Figure S7) and only including stool and swab samples (Supplementary Figure S8). In general, all of the KEGG pathways at Level 1 (Supplementary Table S6**;** Supplementary Figure S9) and Level 2 (Supplementary Table S7) were highly different between the sample types while there were only a few statistically significant differences in abundances of functional categories between participants. This observation is consistent with functional pathways being more similar across individuals than taxonomic assignments (Supplementary Figure S10) as has been observed in other studies^22^.

## Discussion

The primary purpose of this analysis was to determine similarities and differences in the microbial communities of three different types of samples that can be used to assess the gut microbiome: rectal swab samples, stool samples, and normal rectal mucosa samples. This is the first large study to evaluate the longitudinal short-term stability of the microbiota within mucosal biopsies and rectal swabs. We observed distinct differences in the microbiome composition of swab or stool samples for some, but not all, taxa. We found that differences between different sample types extends to functional categorization with whole-genome shotgun sequencing. However, the individual signature of participants was also observed and was largely stable between two time points. Thus, we found that while the distribution of some taxa was associated with these different sampling techniques, other taxa largely reflected individual differences in the microbial community that were insensitive to sampling technique.

A few studies have directly compared the microbiome composition of rectal swabs with rectal mucosal and/or stool samples^12,16,23^. From these and our study, there is increasing evidence that the microbiome composition within the colorectum varies from the mucosa to the lumen, and that swab samples may capture a different microbiome composition profile than either mucosa or stool. However, previous studies reported on small sample sizes. In one such small study (n = 11) of rectal swabs and normal rectal mucosa from individuals with and without a history of colorectal adenoma, bacterial α-diversity was higher in swabs than in mucosa and the swabs also had higher abundances of *Eubacteria* and *Lactobacillus* spp.^16^. In our study, we also found Lactobacillaceae was more abundant in swab than stool. Another study, which compared select phyla in rectal swabs with stool samples in 10 patients with IBD, observed a high intra-subject correlation of Bacteroidetes abundance and Shannon diversity measures while there was little difference by sample type among the subjects, which is consistent with our finding of no statistically significant difference between stool and swab abundances for this phylum^23^. Unlike our study, they did not observe a high correlation in a collective measure of Actinobacteria, Firmicutes, Fusobacteria, and Verrucomicrobia whereas we did find significant differences when these phyla were evaluated individually. In a study of swab and stool samples from 7 pediatric patients and serial stool and rectal mucosa samples from 10 adult patients, differences in taxa were primarily due to differences between mucosa and stool samples with rectal swabs being most similar to the mucosal samples in taxa composition^12^. They further identified 6 genera, which they observed only in mucosa and swab samples and not in stool samples. We observed 3 of 6 of these genera (*Anaerococcus*, *Murdochiella*, and *Peptoniphilus*) to be present in both swab and stool samples in our study which had a larger sample size, although, in general, the relative abundances were lower in stool versus swab. This suggests the importance of sufficient sample sizes to capture rarer taxa regardless of sample type as well as the possibility that the degree to which mucosally associated bacteria slough off into the lumen may vary across individuals due to individual differences or bacterial composition. We also found the similarities between swab and stool samples were highly taxa-specific even within the same phylum or family. Another potential explanation for the small differences between our studies is that the previous study used swab and mucosal samples from different individuals which could have confounded inter-niche differences with inter-person differences. As our study did, this previous study also found evidence of a decreasing aerobic microbiota gradient from the mucosa to the lumen of the colorectum^12^.

In addition to evaluating the sample type, we also considered whether there was variability in the composition over time by evaluating samples collected 3 months apart. We found variability in composition was almost entirely explained by the sample type and inter-person variability indicating that the microbiome composition is relatively stable in all of the sample types during this period of time regardless of season of collection. To our best knowledge, no previous study has evaluated the stability of the microbiota within mucosal biopsies or rectal swabs. However, our findings are largely consistent with other studies which have evaluated repeated stool collections^19,22,40^. In a small study (n = 5) with multiple stool collections across 8 or more years, inter-individual variation was substantially greater than intra-person variability across time although some taxa appeared to have more variation than other taxa^18^. Nonetheless, most of the variation was due to changes in the species abundance versus a presence or absence of the species indicating that an individual-specific microbiota pattern may exist. This was also observed in a study evaluating stool samples collected a year apart (n = 43) in which intra-person variation in the stool metagenome was much smaller than inter-person variability and individual-specific variation patterns over time also remained stable^40^.

It has been reported in several studies that, despite large inter-person differences in the taxa composition of the microbiome, inter-person variability in the gene composition, and, thus the functional activity, of the microbiota is much less^10,18–22^. However, few studies have evaluated differences in the gene composition of the microbiota between samples types within the colorectum. In our study, we found extensive variation in the samples between KEGG pathways due to sample type (stool, swab, rectal mucosa). In a small study of inferred gene content from 16S rRNA analysis comparing rectal swabs (n = 7), stool (n = 28), and rectal mucosa (n = 10) from different individuals, Albenberg *et al*. reported that genes in carbohydrate metabolism are in lower abundance in swab vs. stool or mucosa^12^. We found that the KEGG pathway carbohydrate metabolism was highest in stool and lowest in mucosa with rectal swabs being more similar in relative abundance to stool. This pathway included gene families mapping to enzymes involved in the degradation of colonic mucus such as sialate O-acetylesterase and N-acetylneuraminate lyase. In general, this finding of a relative abundance for swab falling between stool and mucosa was similar for most pathways and gene families, particularly those for metabolism. These findings further indicate the choice of stool, swab, and mucosa samples will measure different aspects of the microbiota within the colorectum.

There are some limitations to our study. We conducted this study among individuals with a history of colorectal polyps. Thus, our findings are generalizable insofar as the gut microbiome characteristics across sample type are consistent between individuals with and without a history of polyps. The sample size, particularly for the metagenomics analysis of colorectal mucosa, is modest and we may thus have had insufficient statistical power to observe differences. However, the comparisons of stool and swab were more robust and the largest to-date. For analysis of the stability of the measurements, samples were collected approximately three months apart and so we could not evaluate stability over a longer time period. However, we were able to capture variability in season of collection. Future studies with multiple repeated collections are needed to further ascertain whether the short-term reliability holds up longitudinally. Lastly, we cannot exclude the possibility of human contamination in the metagenomics analysis of colorectal mucosa.

There are also several strengths to consider. Unlike some previous studies, by including sample types from within the same individual, we were able to evaluate the relative contributions of inter-person vs. inter-niche differences. We were also able to evaluate differences across time by including samples from two time points. Finally, we were able to collect swab and mucosa samples from individuals who had not undergone a bowel cleansing preparation.

## Conclusions

In summary, despite structural differences in the microbial communities being driven largely by the sample type, the individual signature of each participant was still evident and was also stable over time. It thus seems, at least in this southern US population, a single spot sample may be sufficient to capture the microbial signature of an individual. Future studies which compare the microbiome findings with markers of disease are needed to better understand which type of sample may be the most appropriate for the research question.

## Electronic supplementary material

Supplementary Figures and Tables
