# Supplementary material for: Inter-niche and inter-individual variation in gut microbial community assessment using stool, rectal swab, and mucosal samples
Source: Sci Rep. 2018 Mar 7;8:4139. doi: 10.1038/s41598-018-22408-4 (PMC5841359; doi:10.1038/s41598-018-22408-4)
Supplement: Supplementary file 1 — Supplementary Figures and Tables [file 41598_2018_22408_MOESM1_ESM.pdf]

Inter-niche and inter-individual variation in gut microbial community assessment using stool, rectal swab, and mucosal samples

Roshonda B. Jones, Xiangzhu Zhu, Emili Moan, Harvey J. Murff, Reid M. Ness, Douglas L. Seidner, Shan Sun, Chang Yu, Qi Dai, Anthony A. Fodor, M. Andrea Azcarate-Peril, Martha J. Shrubsole

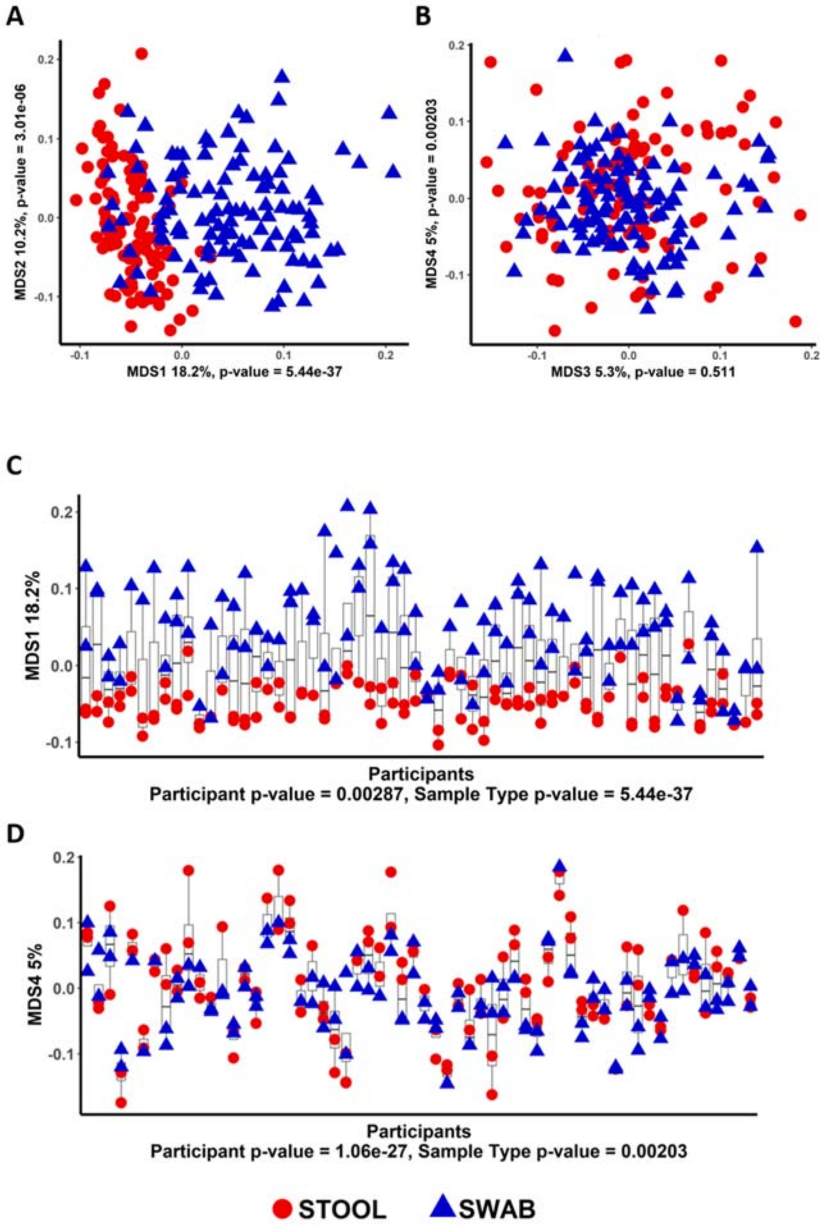

Supplemental Figure S1. Multidimensional scaling (MDS) of rarefied open-reference OTUs classified at the family level using 16S rRNA sequence reads. There are four samples (two each of stool and swab) from each of the 60 participants in our study colored by sample origin (red is stool and blue is swab). Repeated samples collected from individuals were collected with an average separation of 3 months. The distinct separation of colors shows that there is separation by sample type in MDS axis 1 and MDS axis 2 (**A**,**C**) but not in MDS axes 3 and 4 (**B**). However, MDS axis 4 shows strong clustering by participant (**D**).

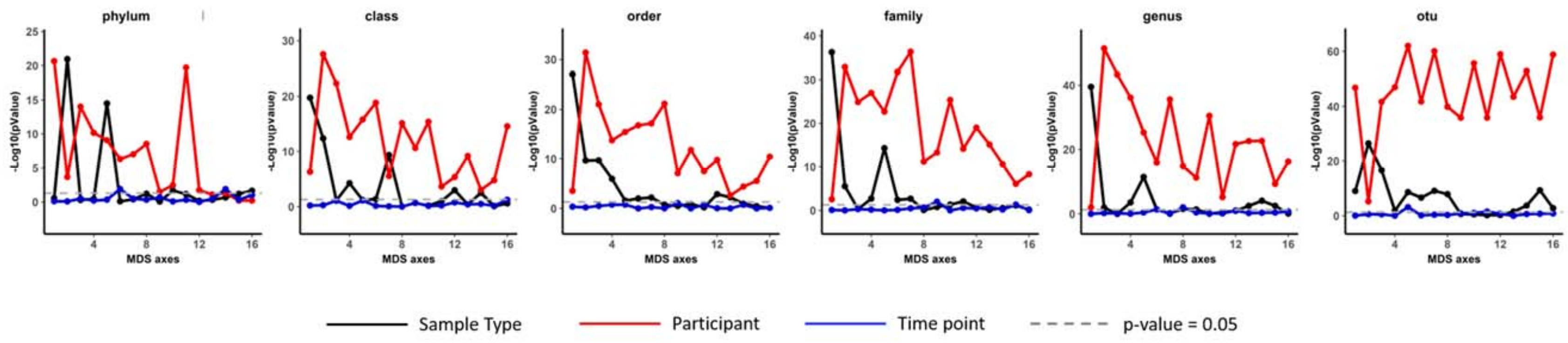

Supplemental FigureS2. The first 15 MDS axes were regressed against sample type (swab or stool), participant ID and time point. The –log10(p-value) for the null hypothesis that sample type, participant ID and time point have no impact on the MDS axes are all shown. While there are significant differences in the first MDS axis in stool vs swab samples, the MDS axes thereafter are significantly different between the participants. Taxonomic calls were based on QIIME rarefied open referenced OTU picking against GreenGenes database.

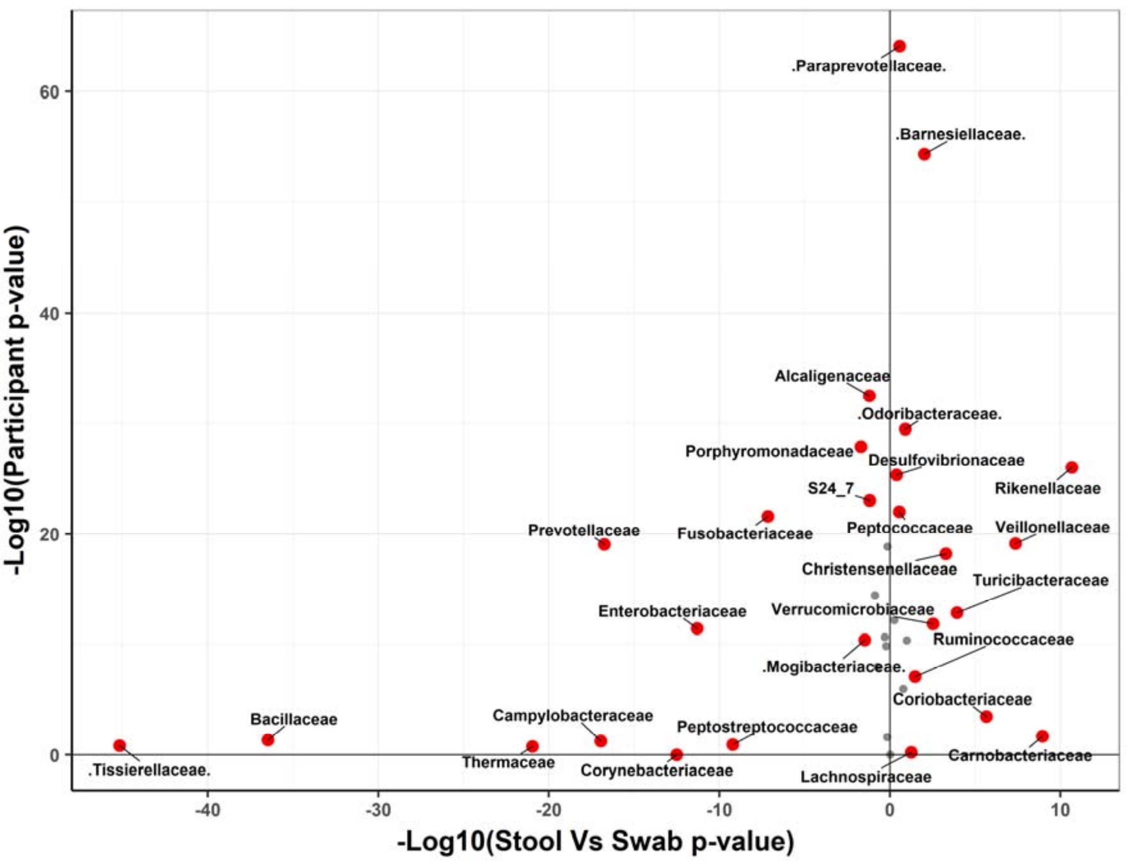

Supplemental Figure S3. For each taxa at the family level present in at least 25% of samples, p-values for a null hypothesis of no difference by stool vs. swab vs. by participant. Red symbols are taxa that have a p-value that is significant at a 10% false discovery rate. Taxa higher in swab than stool have a negative x coordinate and taxa higher in stool than swab have a positive x-coordinate. Data was generated using rarefied open-reference OTUs classified at the family level using 16S rRNA sequence reads.

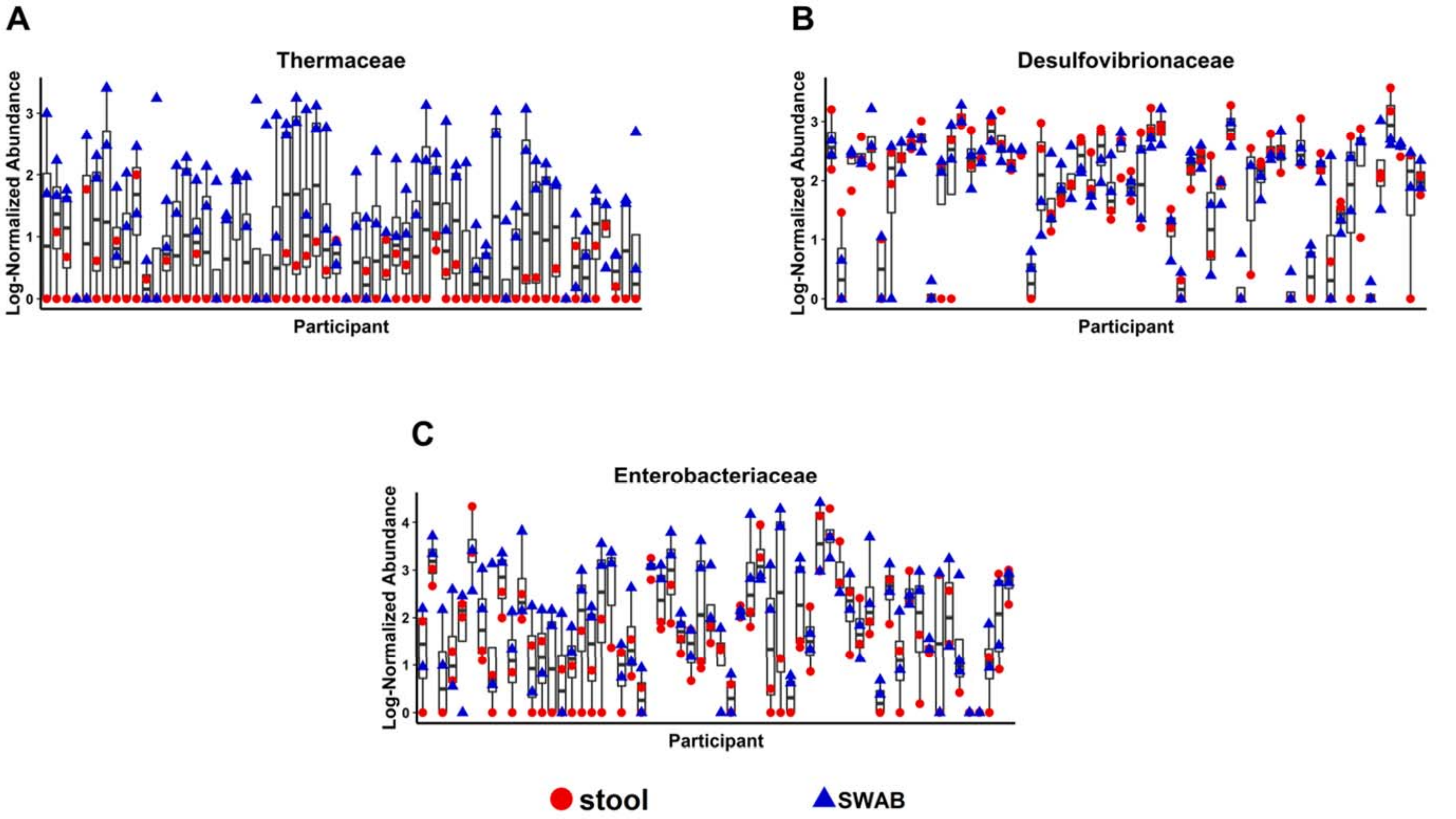

Supplemental Figure S4. Example of taxa at the family level for which the effect on taxa variation is mostly affected by **A**. sample type (stool (red) or swab (blue)), **B**. participant or **C**. both participant and sample type using closed-reference OTUs from 16S rRNA data.

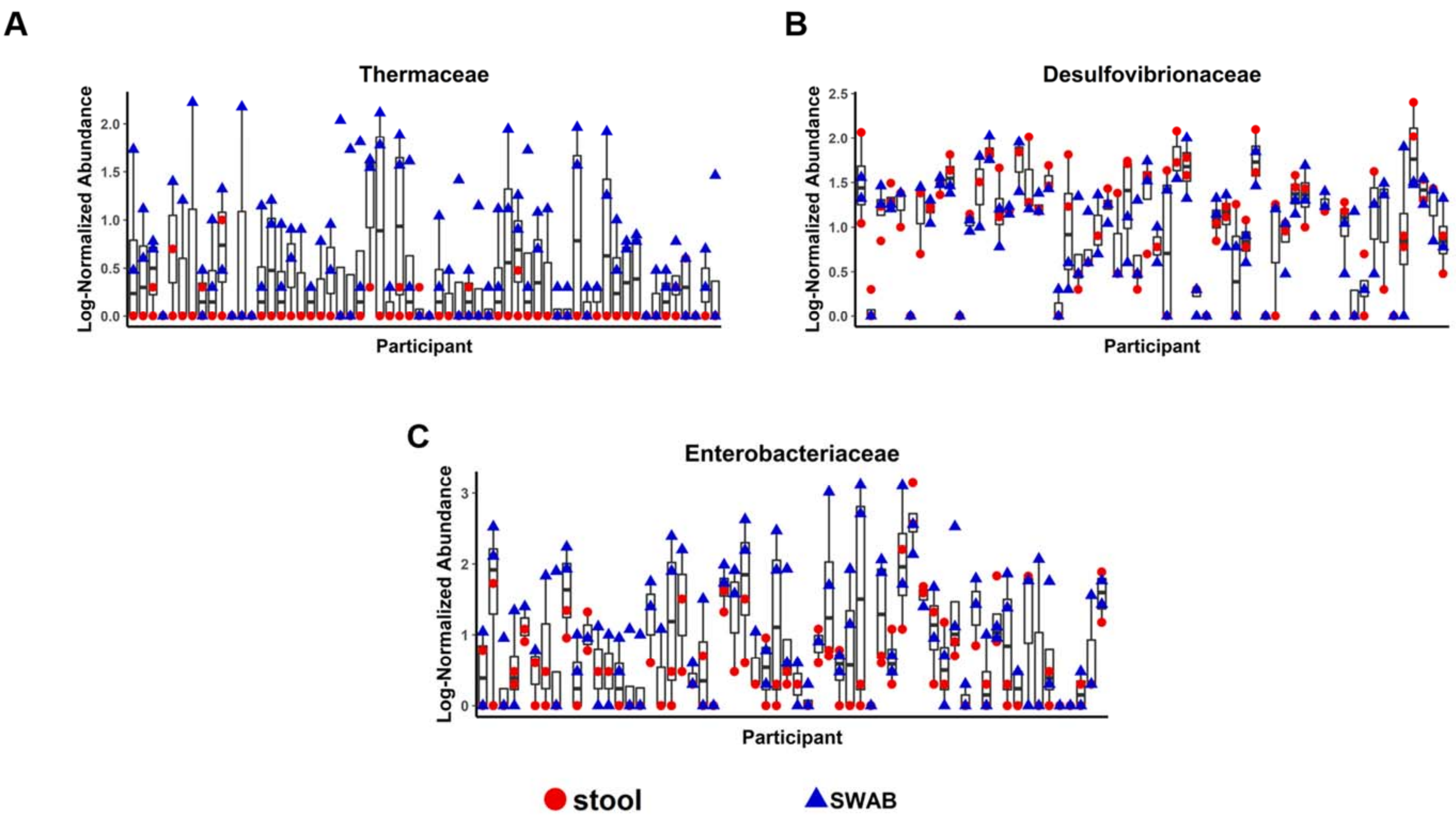

Supplemental Figure S5. Example taxa at the family-level for which the effect on taxa variation is mostly affected by **A**. sample type (stool (red) or swab (blue)), **B**. participant or **C**. both participant and sample type using open-reference OTUs generated from 16S rRNA data.

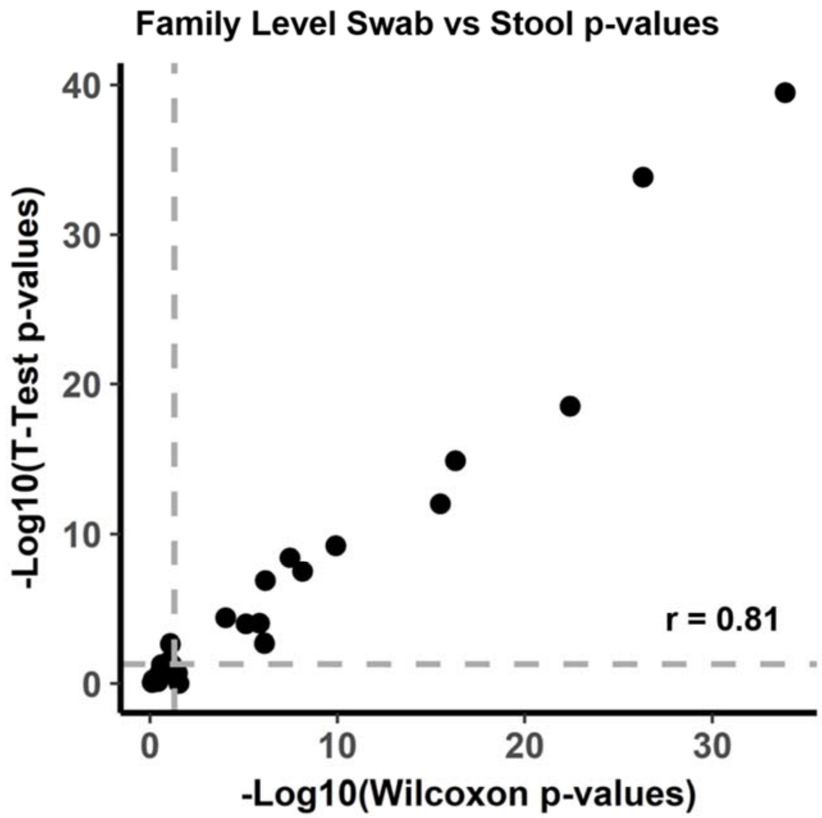

Supplemental Figure S6. Plot of p-values testing differences in family level 16S rRNA abundances of swab and stool samples generated using T-tests (parametric) and Wilcoxon tests (non-parametric). Data was generated using rarefied open-reference OTUs classified at the family level using 16S rRNA sequence reads.

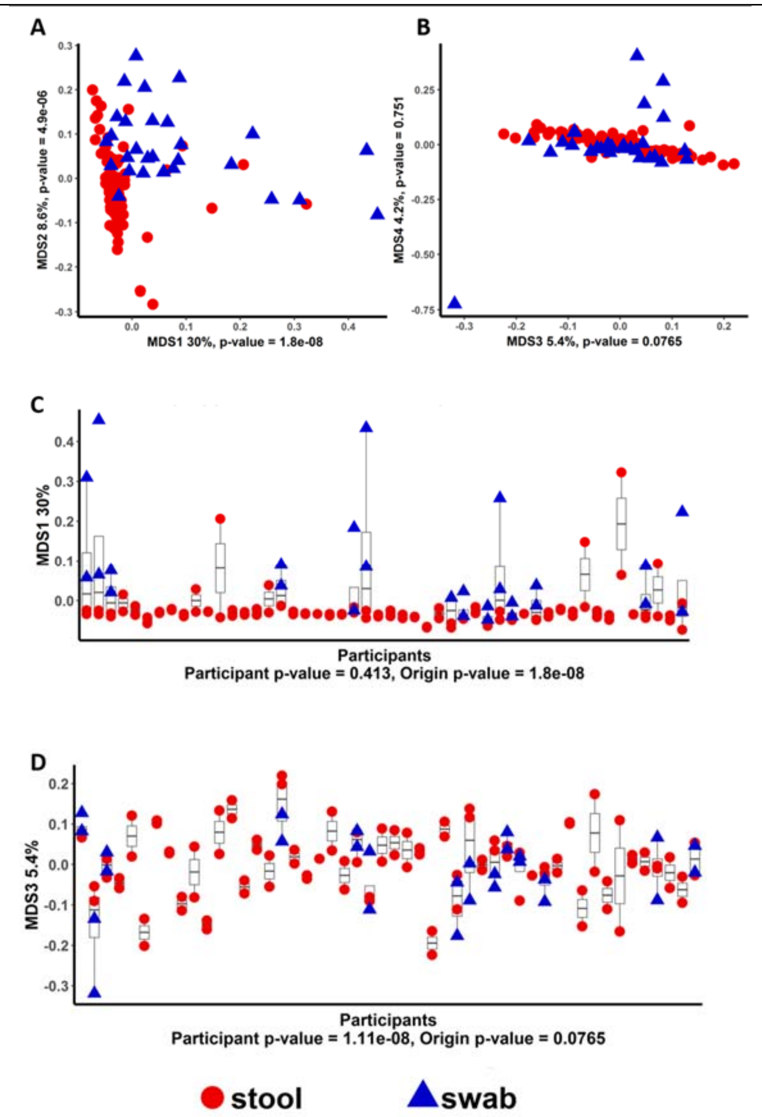

Supplemental Figure S7. Plot of first two coordinates of an MDS ordination of the KEGG gene family abundance table for WGS sequence reads from only swab (blue triangles), stool (red circles) samples. The distinct separation of colors shows that there is separation by sample type in MDS axis 1, and MDS axis 2 (**A**, **C**) and by sample type in MDS 3 or MDS4 (**B**). However, variation in MDS axis 3 is explained by participant which is evidenced in the differences in the means of the bar plots (**D**).

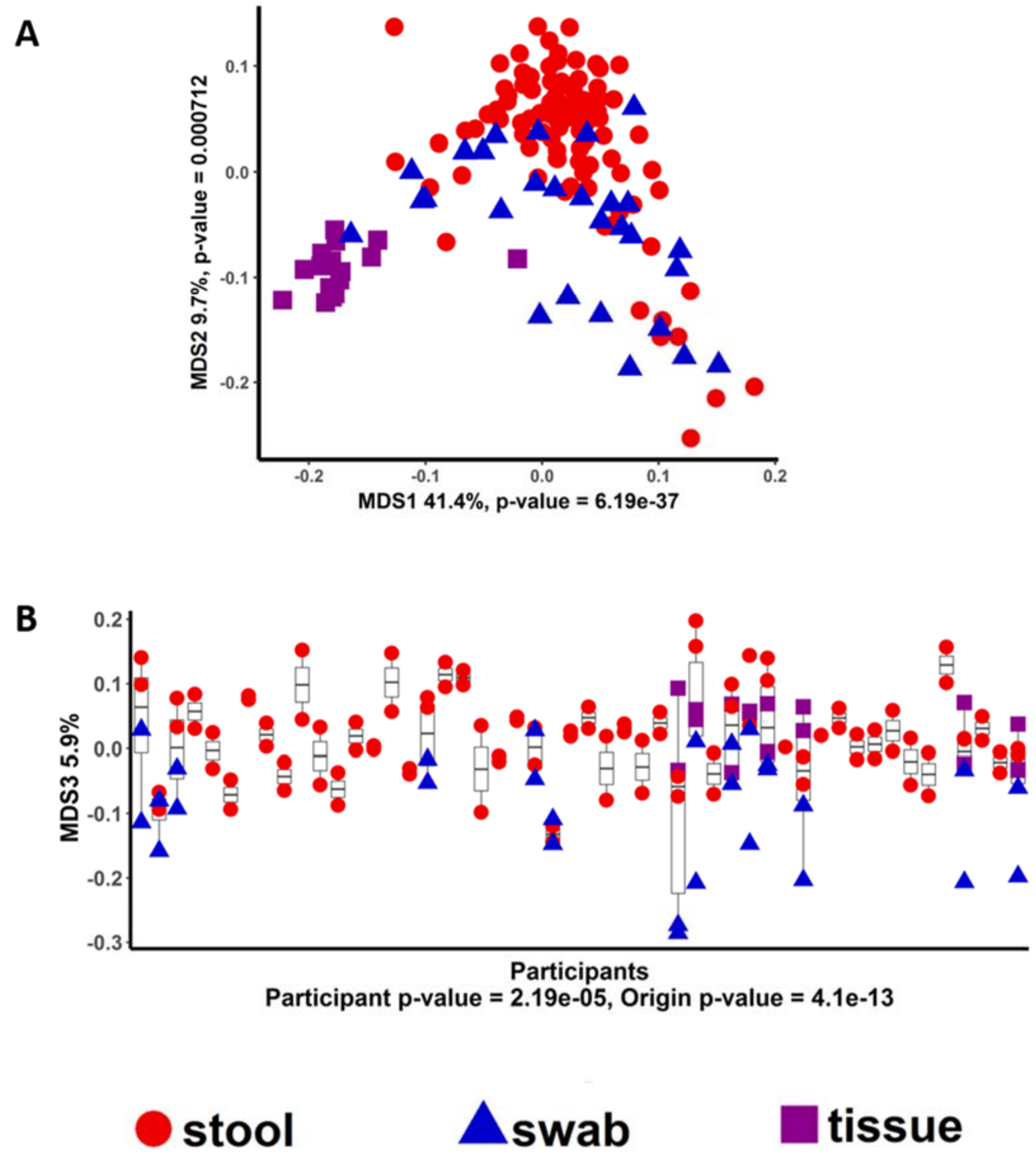

Supplemental Figure S8. Plot of first two coordinates of an MDS ordination of the WGS sequence reads classified into family taxonomic levels for only swab samples (blue triangles) stool samples (red circles), and tissue samples (purple squares). The first axis and second axes show sample type separation (**A**). The third axis shows strong participant variation as well as separation by sample type (**B**)

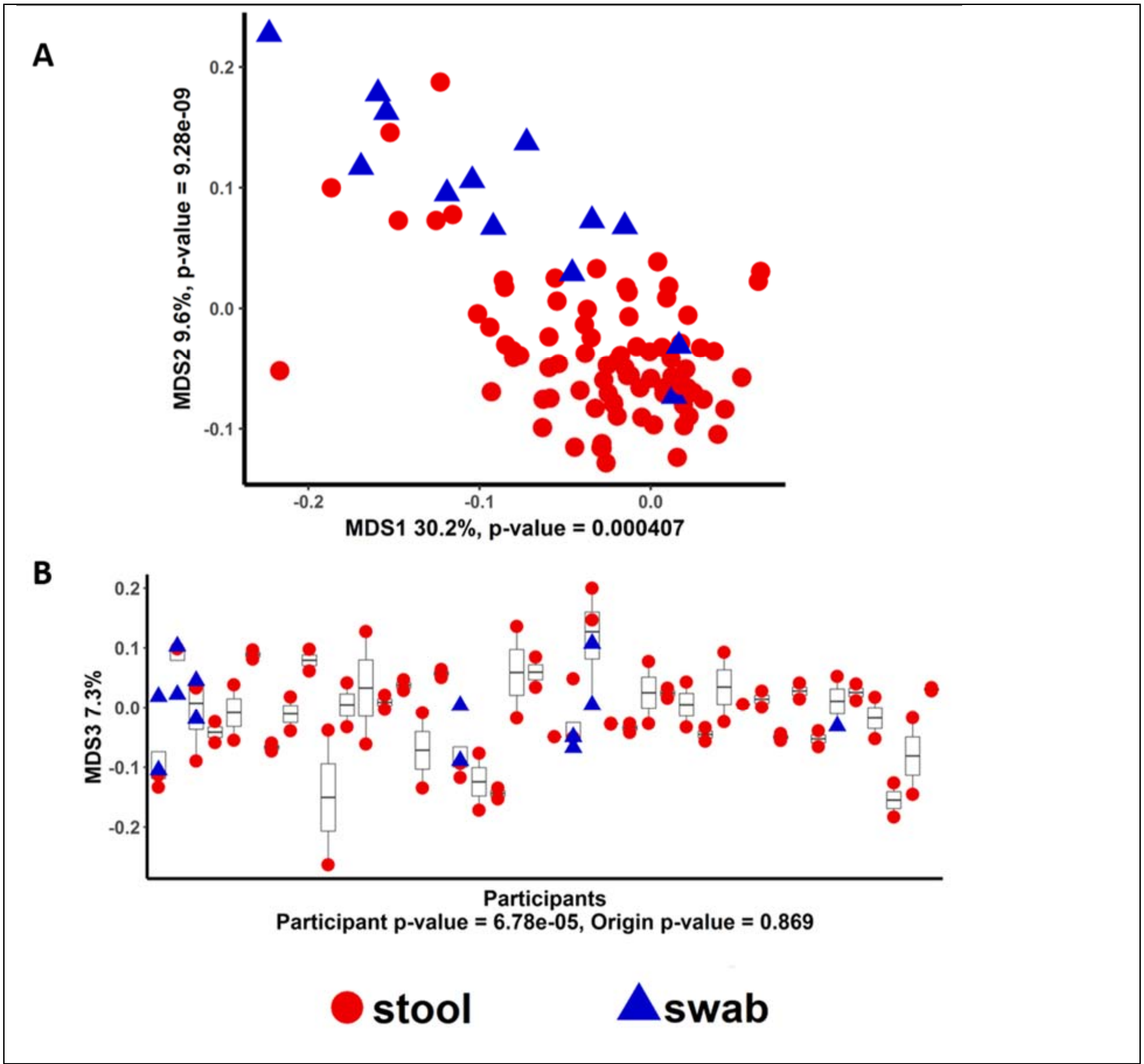

Supplemental Figure S9. Plot of first two coordinates of an MDS ordination of the WGS sequence reads classified into family taxonomic levels for only swab samples (blue triangles), and stool samples (red circles)). The first axis and second axes show sample origin separation (**A**). The third axis shows strong participant variation (**B**).

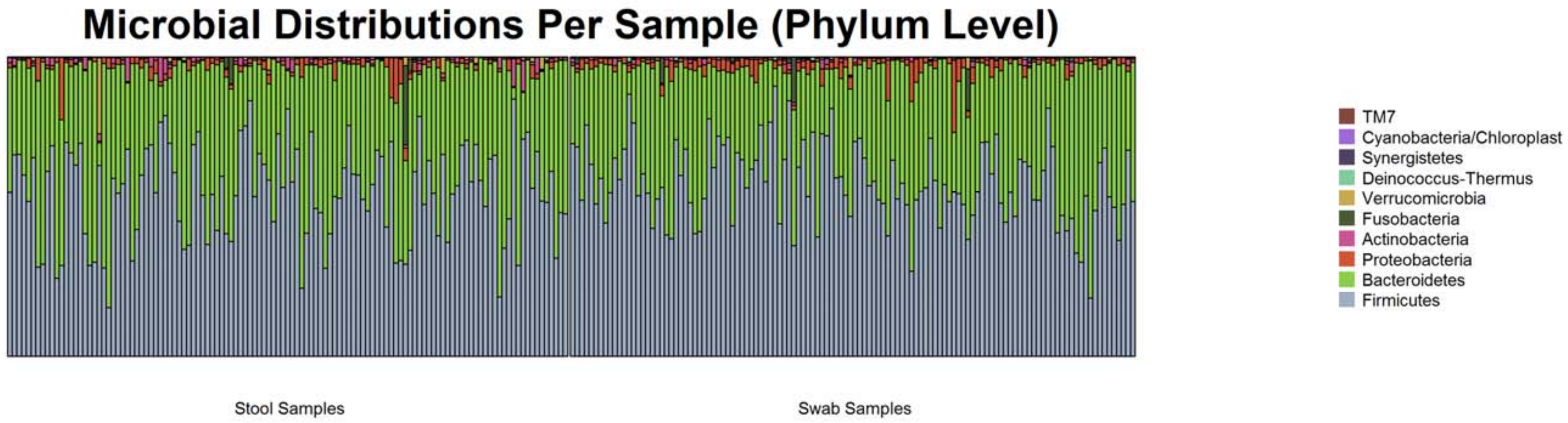

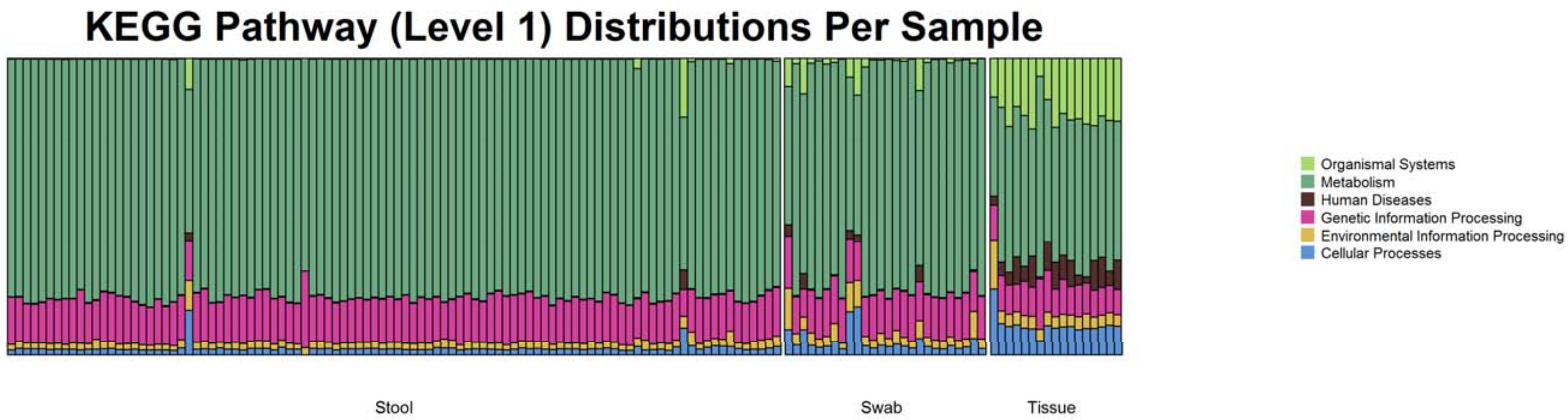

Supplemental Figure S10. Distribution of RDP calls at the phyla level (A) and distribution of KEGG gene pathways at the highest level (B). The distributions of RDP calls vary greatly between samples. However, in general, the distributions of KEGG pathway distributions for stool samples have little variation.

Supplemental Table S1. Characteristics of study participants.

Characteristics	Overall	16S rRNA amplicon sequencing participants	WGS participants		
	n=68	n=60	n=50^	n=14*	n=8**
Age (years), mean±SD	62.7±7.5	62.5±7.5	63.1±7.3	65.0±7.8	64.20±7.6
Male, n (%)	39 (57.3)	32 (53.3)	29 (58.0)	9 (64.3)	7 (87.5)
White, n (%)	68 (100)	60 (100)	50 (100)	14 (100)	8 (100)
Smoking status, n (%)					
Never	33 (48.5)	31 (51.7)	24 (48.0)	5 (35.7)	2 (25.0)
Former	30 (44.1)	24 (40.0)	22 (44.0)	9 (64.3)	6 (75.0)
Current	5 (7.4)	5 (8.3)	4 (8.0)		
Alcohol drinking status, n (%)					
Never	25 (36.8)	21 (35.0)	21 (42.0)	7 (50.0)	4 (50.0)
Former	12 (17.6)	11 (18.3)	7 (14.0)	1 (7.1)	1 (12.5)
Current	31 (45.6)	28 (46.7)	22 (44.0)	6 (42.9)	3 (37.5)
Body mass index (kg/m^2), mean±SD	29.6±5.3	29.3±5.1	29.6±5.4	31.0±5.5	31.6±6.1
Thr1482Ile polymorphism, n (%)					
GG	49 (72.1)	42 (70.0)	31 (62.0)	10 (71.4)	7 (87.5)
GA/AA	19 (27.9)	18 (30.0)	19 (38.0)	4 (28.6)	1 (12.5)
Treatment arm, %	35 (51.4)	31 (51.7)	24 (48.0)	6 (42.8)	4 (50.0)
Baseline sample collection season, n (%)					
Spring	16 (23.5)	16 (26.7)	12 (24.0)	1 (7.1)	
Summer	30 (44.1)	29 (48.3)	13 (26.0)	4 (28.6)	1 (12.5)
Autumn	12 (17.7)	7 (11.7)	15 (30.0)	5 (35.7)	5 (62.5)
Winter	10 (14.7)	8 (13.3)	10 (20.0)	4 (28.6)	2 (25.0)
Time between clinic visits (days), mean±SD	86.4±6.6	86.4±6.9	85.4±5.4	83.9±4.6	86.1±4.0

WGS: Whole-genome shotgun metagenomics DNA sequencing

^ Stool samples were analyzed

* Both stool and swab samples were analyzed

** Stool, swab, and mucosa samples were analyzed

Supplemental Table S2. Differences in MDS axes of rarefied open-reference OTUs of 16S rRNA gene sequence read counts classified to family level taxa due to sample source (stool vs. swab) and participant source.

Item	Mean ± Standard Deviation		Stool vs. Swab p value[a]	Participant p value[c]	R-squared Marginal[d]	R-squared Conditional[d]
	Stool	Swab				
**Analysis of MDS axes**						
Sun rarified Richness	9.74 ± 1.7	11.10 ± 2.2	4.63×10^{-09}	3.77×10^{-08}	0.103	0.429
Shannon diversity	1.76 ± 0.3	1.89 ± 0.3	2.34×10^{-05}	9.68×10^{-11}	0.048	0.462
Shannon evenness	0.54 ± 0.1	0.56 ± 0.1	0.01	1.18×10^{-07}	0.02	0.37
MDS axis 1	-0.048 ± 0.025	0.048 ± 0.061	8.16×10^{-36}	2.87×10^{-03}	0.514	0.606
MDS axis 2	-0.01 ± 0.071	0.009 ± 0.062	1.51×10^{-05}	9.98×10^{-33}	0.028	0.747
MDS axis 3	0.001 ± 0.074	-0.001 ± 0.061	0.59	3.28×10^{-25}	0.001	0.651
MDS axis 4	0.008 ± 0.077	-0.008 ± 0.056	6.27×10^{-03}	3.98×10^{-27}	0.014	0.697
MDS axis 5	-0.024 ± 0.068	0.024 ± 0.059	3.87×10^{-14}	4.37×10^{-23}	0.115	0.673
MDS axis 6	-0.007 ± 0.069	0.007 ± 0.065	9.55×10^{-03}	8.75×10^{-32}	0.012	0.717
MDS axis 7	-0.006 ± 0.074	0.007 ± 0.061	6.27×10^{-03}	6.71×10^{-36}	0.012	0.761
MDS axis 8	0 ± 0.071	-0.001 ± 0.065	0.85	8.28×10^{-12}	0.004	0.445
MDS axis 9	-0.005 ± 0.061	0.004 ± 0.073	0.27	7.23×10^{-14}	0.021	0.492
MDS axis 10	-0.005 ± 0.066	0.004 ± 0.069	0.09	1.26x10-25	0.007	0.654
MDS axis 11	0.009 ± 0.063	-0.01 ± 0.07	0.02	1.14×10^{-14}	0.019	0.506
MDS axis 12	-0.002 ± 0.072	0.002 ± 0.064	0.29	1.85×10^{-19}	0.005	0.587
MDS axis 13	-0.002 ± 0.063	0.002 ± 0.072	0.80	1.32×10^{-15}	0.004	0.532
MDS axis 14	0.002 ± 0.069	-0.002 ± 0.067	0.37	3.05×10^{-11}	0.004	0.452
MDS axis 15	-0.007 ± 0.069	0.008 ± 0.065	0.12	8.39×10^{-07}	0.022	0.344

[a] p-value derived from ANOVA of the mixed linear model

Supplemental Table S3. Differences in rarefied open-reference OTUs 16S rRNA gene sequence read counts classified to family level taxa due to sample source (stool vs. swab) and participant source.

Family Level Taxa[a]	Log-Normalized Mean Abundance ± Standard deviation		Stool vs. Swab p value[b]	Participant p value[c]	R-squared Marginal[d]	R-squared Conditional[d]
	Stool	Swab				
[Barnesiellaceae]	1.123 ± 0.912	1.023 ± 0.887	0.020894	9.21E-54	0.006	0.863
[Mogibacteriaceae]	0.899 ± 0.523	1.045 ± 0.591	0.06253	6.68E-11	0.012	0.446
[Odoribacteraceae]	1.301 ± 0.632	1.262 ± 0.621	0.185	3.07E-29	0.003	0.698
[Paraprevotellacea]	0.589 ± 0.966	0.517 ± 0.814	0.3441	2.77E-63	0.001	0.901
[Tissierellaceae]	0.173 ± 0.226	1.725 ± 0.804	2.50E-44	0.159212	0.633	0.666
Actinomycetaceae	0.469 ± 0.386	0.492 ± 0.566	0.729429	0.030748	0.007	0.15
Alcaligenaceae	1.557 ± 0.847	1.634 ± 0.779	0.102423	3.79E-32	0.011	0.728
Bacillaceae	0.722 ± 0.432	2.177 ± 0.869	6.31E-36	0.05365	0.536	0.595
Bacteroidaceae	3.427 ± 0.292	3.371 ± 0.29	0.15725	7.00E-11	0.011	0.444
Campylobacteraceae	0.041 ± 0.128	0.72 ± 0.762	1.02E-16	0.065884	0.275	0.365
Carnobacteriaceae	0.205 ± 0.281	0.036 ± 0.113	4.26E-09	0.025504	0.141	0.274
Christensenellaceae	0.755 ± 0.648	0.594 ± 0.568	0.001322	1.70E-18	0.027	0.59
Clostridiaceae	1.522 ± 0.593	1.488 ± 0.544	0.611061	1.38E-12	0.002	0.473
Comamonadaceae	0.296 ± 0.515	0.311 ± 0.504	0.741028	4.07E-19	0.001	0.576
Coriobacteriaceae	1.61 ± 0.525	1.355 ± 0.363	6.23E-06	0.000447	0.08	0.294
Corynebacteriaceae	0.006 ± 0.041	0.427 ± 0.549	1.90E-12	1	0.225	0.225
Desulfovibrionaceae	0.995 ± 0.653	0.956 ± 0.59	0.496516	2.39E-25	0.001	0.664
Enterobacteriaceae	0.534 ± 0.645	1.1 ± 0.877	2.57E-11	6.97E-12	0.128	0.511
Erysipelotrichaceae	1.927 ± 0.575	2.018 ± 0.39	0.190692	8.98E-15	0.01	0.516
Fusobacteriaceae	0.215 ± 0.627	0.505 ± 0.751	2.11E-07	9.07E-22	0.051	0.663
Gemellaceae	0.171 ± 0.279	0.169 ± 0.413	0.971	1	0	0
Lachnospiraceae	3.281 ± 0.195	3.224 ± 0.249	0.09761	0.623714	0.042	0.072
Lactobacillaceae	0.28 ± 0.435	0.305 ± 0.444	0.572344	4.09E-11	0.001	0.422
Pasteurellaceae	0.212 ± 0.418	0.158 ± 0.35	0.227481	1.62E-06	0.007	0.339
Peptococcaceae	0.424 ± 0.478	0.411 ± 0.488	0.3441	3.75E-22	0.013	0.633
Peptostreptococcaceae	0.063 ± 0.153	0.477 ± 0.669	2.49E-09	0.134125	0.154	0.234
Porphyromonadaceae	1.588 ± 0.83	1.759 ± 0.769	0.040494	8.73E-28	0.01	0.685
Prevotellaceae	0.748 ± 1.053	1.671 ± 1.06	1.30E-16	2.69E-19	0.15	0.653
Rikenellaceae	2.18 ± 0.612	1.848 ± 0.643	1.01E-10	5.85E-26	0.077	0.683
Ruminococcaceae	3.175 ± 0.294	3.115 ± 0.307	0.06253	1.22E-07	0.015	0.378
S24_7	0.313 ± 0.668	0.434 ± 0.742	0.104083	4.35E-23	0.007	0.639
Streptococcaceae	1.577 ± 0.687	1.682 ± 0.67	0.2405	1.65E-08	0.005	0.386
Thermaceae	0.045 ± 0.152	0.717 ± 0.644	1.36E-20	0.183912	0.348	0.404
Turicibacteraceae	0.316 ± 0.548	0.137 ± 0.26	0.000309	3.08E-13	0.042	0.511
Unassigned	2.839 ± 0.293	2.865 ± 0.295	0.651853	2.32E-10	0.001	0.443

Family Level Taxa[a]	Log-Normalized Mean Abundance ± Standard deviation		Stool vs. Swab p value[b]	Participant p value[c]	R-squared Marginal[d]	R-squared Conditional[d]
	Stool	Swab				
Veillonellaceae	2.362 ± 0.505	2.094 ± 0.501	1.46E-07	2.44E-19	0.061	0.614
Verrucomicrobiaceae	0.771 ± 0.854	0.552 ± 0.647	0.006984	2.86E-12	0.026	0.479

[a] Limited to families present in at least 25% of samples [b] Benjamini-Hochberg corrected p-value derived from ANOVA of mixed linear model [c] Benjamini-Hochberg corrected p-value derived from ANOVA of linear models with and without participant as a random effect [d] R-squared marginal represents the variation that is explained by the model without the mixed effect (participant) while the conditional R-squared represents the variation that is explained by the model including both fixed effects and mixed effects

Supplemental Table S4. P-values for differences in swab and stool samples for rarefied open-reference OTUs of 16S rRNA gene sequence read counts at the genus level.

Bacteria (Genus level)	Sample Type			Participant	Culture Medium
	Stool Mean	Swab Mean	Adj. p-value	Adj. p-value	
**Peptoniphilus**	0.573	2.504	$2.00X10^{-44}$	$8.77x10^{-03}$	Anaerobe
**Anaerococcus**	0.285	2.023	$2.01X10^{-42}$	$7.17X10^{-03}$	Anaerobe
**Finegoldia**	0.307	2.076	$5.32X10^{-40}$	$1.74X10^{-01}$	Anaerobe
**Anoxybacillus**	1.327	2.904	$3.12X10^{-35}$	$5.53X10^{-03}$	Aerobe
**Thermus**	0.208	1.576	$1.29X10^{-32}$	$1.11X10^{-01}$	Anaerobe
**Geobacillus**	1.635	3.120	$7.36X10^{-32}$	$3.11X10^{-01}$	Aerobe
**Anaerosphaera**	0.057	1.281	$2.50X10^{-31}$	$1.18X10^{-02}$	Anaerobe
**Porphyromonas**	0.458	1.800	$3.24X10^{-27}$	$2.90X10^{-06}$	Anaerobe
**Acinetobacter**	0.067	0.978	$1.56X10^{-22}$	$1.29X10^{-01}$	Aerobe
**Campylobacter**	0.332	1.479	$1.76X10^{-22}$	$1.57X10^{-05}$	Aerobe
**Murdochiella**	0.120	1.257	$3.64X10^{-22}$	$2.06x10^{-03}$	Anaerobe
**Prevotella**	1.714	2.829	$7.93X10^{-22}$	$6.75X10^{-22}$	Anaerobe
**Negativicoccus**	0.013	0.747	$4.76X10^{-18}$	$5.90X10^{-02}$	Anaerobe
**Asaccharobacter**	1.372	0.732	$1.00X10^{-15}$	$3.79X10^{-17}$	Anaerobe
**Peptostreptococcus**	0.183	1.010	$4.70X10^{-14}$	$1.04X10^{-04}$	Anaerobe
**Corynebacterium**	0.104	0.826	$8.88X10^{-14}$	$3.15X10^{-01}$	Aerobe
**Escherichia_Shigella**	0.992	1.829	$9.88X10^{-13}$	$2.12X10^{-12}$	Aerobe
**Granulicatella**	0.776	0.264	$4.19X10^{-12}$	$4.44X10^{-02}$	Aerobe
**Varibaculum**	0.008	0.466	$1.73X10^{-11}$	$3.41X10^{-01}$	Aerobe
**Peptococcus**	0.154	0.616	$3.56X10^{-11}$	$2.50X10^{-06}$	Anaerobe
**Alicyclobacillus**	0.028	0.439	$1.23X10^{-10}$	$2.58X10^{-01}$	Aerobe
**Fusobacterium**	0.347	0.991	$1.23X10^{-10}$	$5.61X10^{-08}$	Anaerobe
**Fusibacter**	1.391	0.913	$9.69X10^{-10}$	$1.02X10^{-27}$	Anaerobe
**Mobiluncus**	0.054	0.553	$1.95X10^{-09}$	$1.60X10^{-01}$	Anaerobe
**Gordonibacter**	1.411	0.855	$4.23X10^{-09}$	$2.78X10^{-09}$	Anaerobe

Bacteria (Genus level)	Sample Type			Participant	Culture Medium
	Stool Mean	Swab Mean	Adj. p-value	Adj. p-value	
**Dialister**	1.417	2.090	$8.83X10^{-09}$	$1.61X10^{-18}$	Anaerobe
**Streptophyta**	0.745	0.250	$1.02X10^{-08}$	$1.58X10^{-01}$	Unknown
**Roseburia**	3.425	3.151	$1.87X10^{-07}$	$3.45X10^{-10}$	Anaerobe
**Clostridium_XlVa**	3.181	3.389	$4.79X10^{-07}$	$1.65X10^{-17}$	Anaerobe
**Anaerofustis**	0.611	0.272	$5.76X10^{-07}$	$6.48x10^{-04}$	Anaerobe
**Alistipes**	3.346	2.988	$1.71X10^{-06}$	$2.88X10^{-19}$	Anaerobe
**Dorea**	3.150	2.906	$3.52X10^{-06}$	$2.50X10^{-06}$	Anaerobe
**Hallella**	0.230	0.680	$4.93X10^{-06}$	$5.87X10^{-11}$	Anaerobe
**Brevibacillus**	0.066	0.349	$1.14X10^{-05}$	$6.26X10^{-01}$	Aerobe
**Lachnospiracea_incertae_sedis**	4.089	3.902	$3.54X10^{-05}$	$8.40X10^{-06}$	Anaerobe
**Salmonella**	0.142	0.388	$9.96X10^{-05}$	$2.11X10^{-11}$	Aerobe
**Aminiphilus**	0.309	0.146	$4.92X10^{-04}$	$2.02X10^{-16}$	Anaerobe
**Clostridium_sensu_stricto**	1.410	1.014	$5.22X10^{-04}$	$1.33X10^{-16}$	Anaerobe
**Veillonella**	1.350	1.057	$5.93X10^{-04}$	$2.69X10^{-22}$	Anaerobe
**Parvimonas**	0.153	0.453	$9.08X10^{-04}$	$3.36X10^{-03}$	Anaerobe
**Acetivibrio**	0.516	0.308	$1.52X10^{-03}$	$1.04X10^{-18}$	Anaerobe
**Ethanoligenens**	0.945	0.695	$1.85X10^{-03}$	$2.36X10^{-16}$	Anaerobe
**Turicibacter**	0.834	0.552	$2.15X10^{-03}$	$1.08X10^{-12}$	Anaerobe
**Phascolarctobacterium**	2.673	2.419	$2.22X10^{-03}$	$1.94X10^{-36}$	Anaerobe
**Coprobacillus**	1.308	1.619	$2.41X10^{-03}$	$3.72X10^{-16}$	Anaerobe
**Lactococcus**	1.043	0.731	$2.76X10^{-03}$	$2.47X10^{-04}$	Aerobe
**Actinomyces**	1.315	1.004	$3.39X10^{-03}$	$1.07X10^{-01}$	Aerobe
**Enterobacter**	0.497	0.779	$3.85X10^{-03}$	$6.23X10^{-12}$	Aerobe
**Rothia**	0.420	0.239	$4.35X10^{-03}$	$1.05X10^{-08}$	Aerobe
**Bifidobacterium**	0.422	0.252	$4.72X10^{-03}$	$4.91X10^{-10}$	Anaerobe
**Megasphaera**	0.318	0.544	$5.61X10^{-03}$	$1.95X10^{-23}$	Anaerobe
**Sporobacter**	0.522	0.354	$6.70X10^{-03}$	$1.16X10^{-15}$	Anaerobe
**Akkermansia**	1.553	1.200	$7.52X10^{-03}$	$1.50X10^{-14}$	Anaerobe
**Clostridium_IV**	3.046	2.864	$7.67X10^{-03}$	$6.92X10^{-11}$	Anaerobe
**Anaerostipes**	1.826	1.586	$8.88X10^{-03}$	$1.48X10^{-06}$	Anaerobe

Bacteria (Genus level)	Sample Type			Participant	Culture Medium
	Stool Mean	Swab Mean	Adj. p-value	Adj. p-value	
**Erysipelotrichaceae_incertae_sedis**	2.148	2.329	$1.11X10^{-02}$	$4.95X10^{-27}$	Aerobe
**Slackia**	0.606	0.444	$1.90X10^{-02}$	$5.39X10^{-34}$	Anaerobe
**Clostridium_XI**	1.894	1.662	$2.22X10^{-02}$	$1.65X10^{-15}$	Anaerobe
**Allisonella**	0.149	0.268	$3.18X10^{-02}$	$1.81X10^{-03}$	Aerobe
**Parasutterella**	2.145	2.337	$6.32X10^{-02}$	$7.71X10^{-44}$	Anaerobe
**Acetanaerobacterium**	1.169	1.017	$6.81X10^{-02}$	$8.68X10^{-03}$	Anaerobe
**Subdoligranulum**	3.002	2.893	$7.49X10^{-02}$	$3.82X10^{-37}$	Anaerobe
**Marvinbryantia**	0.434	0.567	$9.11X10^{-02}$	$2.11X10^{-11}$	Anaerobe
**Collinsella**	2.231	2.075	$9.67X10^{-02}$	$2.11X10^{-29}$	Anaerobe
**Gemella**	0.698	0.509	$9.67X10^{-02}$	$2.58X10^{-01}$	Aerobe
**Allobaculum**	0.521	0.662	$1.08X10^{-01}$	$1.47X10^{-14}$	Anaerobe
**Bacteroides**	4.554	4.507	$1.25X10^{-01}$	$1.68X10^{-12}$	Anaerobe
**Atopobium**	0.348	0.487	$1.26X10^{-01}$	$1.58X10^{-01}$	Aerobe
**Gemmiger**	0.955	0.878	$1.26X10^{-01}$	$1.15X10^{-38}$	Anaerobe
**Lactonifactor**	0.597	0.496	$1.26X10^{-01}$	$5.05X10^{-04}$	Anaerobe
**Robinsoniella**	0.272	0.373	$1.38X10^{-01}$	$1.87X10^{-05}$	Anaerobe
**Enterococcus**	0.294	0.384	$1.39X10^{-01}$	$7.89X10^{-27}$	Aerobe
**Anaerotruncus**	2.410	2.278	$1.57X10^{-01}$	$5.13X10^{-15}$	Anaerobe
**Clostridium_XlVb**	2.597	2.701	$1.57X10^{-01}$	$1.29X10^{-14}$	Anaerobe
**Propionibacterium**	0.164	0.235	$1.62X10^{-01}$	$4.08X10^{-05}$	Anaerobe
**Pseudoflavonifractor**	2.430	2.518	$1.97X10^{-01}$	$8.08X10^{-06}$	Anaerobe
**Paraprevotella**	1.887	1.789	$2.07X10^{-01}$	$2.03X10^{-29}$	Anaerobe
**Oribacterium**	0.268	0.200	$2.20X10^{-01}$	$1.76X10^{-04}$	Anaerobe
**Sutterella**	0.950	1.040	$2.20X10^{-01}$	$2.53X10^{-37}$	Anaerobe
**Desulfovibrio**	0.867	0.760	$2.25X10^{-01}$	$3.27X10^{-36}$	Anaerobe
**Butyrivibrio**	0.825	0.722	$2.50X10^{-01}$	$2.56X10^{-15}$	Anaerobe
**Succinispira**	0.325	0.405	$2.57X10^{-01}$	$6.04X10^{-36}$	Anaerobe
**Parasporobacterium**	0.192	0.140	$2.73X10^{-01}$	$3.91X10^{-05}$	Anaerobe
**Ruminococcus**	2.645	2.545	$2.85X10^{-01}$	$4.79X10^{-27}$	Anaerobe
**Blautia**	3.633	3.569	$2.86X10^{-01}$	$8.24X10^{-02}$	Anaerobe

Bacteria (Genus level)	Sample Type			Participant	Culture Medium
	Stool Mean	Swab Mean	Adj. p-value	Adj. p-value	
**Butyricicoccus**	2.612	2.561	$3.57X10^{-01}$	$5.85X10^{-05}$	Anaerobe
**Eubacterium**	0.383	0.305	$3.63X10^{-01}$	$3.60X10^{-06}$	Anaerobe
**Bilophila**	1.573	1.639	$3.73X10^{-01}$	$1.05X10^{-28}$	Anaerobe
**Acidaminococcus**	0.409	0.469	$3.78X10^{-01}$	$2.60X10^{-34}$	Anaerobe
**Klebsiella**	0.381	0.307	$4.23X10^{-01}$	$3.47X10^{-27}$	Aerobe
**Ralstonia**	0.427	0.493	$4.53X10^{-01}$	$2.13X10^{-02}$	Aerobe
**Streptococcus**	2.736	2.800	$4.57X10^{-01}$	$1.09X10^{-11}$	Aerobe
**Megamonas**	0.226	0.267	$4.59X10^{-01}$	$4.62X10^{-43}$	Anaerobe
**Hydrogenoanaerobacterium**	0.725	0.660	$4.80X10^{-01}$	$7.55X10^{-11}$	Anaerobe
**Anaerovorax**	1.997	1.949	$5.20X10^{-01}$	$6.02X10^{-11}$	Anaerobe
**Catenibacterium**	0.504	0.438	$5.20X10^{-01}$	$2.50X10^{-31}$	Anaerobe
**Cloacibacillus**	0.273	0.240	$5.37X10^{-01}$	$7.19X10^{-39}$	Anaerobe
**Clostridium_XVIII**	2.603	2.559	$5.44X10^{-01}$	$2.07X10^{-10}$	Anaerobe
**Faecalibacterium**	3.567	3.630	$5.65X10^{-01}$	$7.12X10^{-36}$	Anaerobe
**Mogibacterium**	0.711	0.676	$5.86X10^{-01}$	$1.68X10^{-14}$	Anaerobe
**Parabacteroides**	3.362	3.324	$5.86X10^{-01}$	$5.08X10^{-38}$	Anaerobe
**Haemophilus**	0.539	0.491	$5.89X10^{-01}$	$2.21X10^{-09}$	Aerobe
**Sphingomonas**	0.141	0.168	$5.90X10^{-01}$	$1.54X10^{-06}$	Aerobe
**Lactobacillus**	0.877	0.941	$6.34X10^{-01}$	$3.66X10^{-05}$	Anaerobe
**Clostridium_XIX**	0.377	0.390	$6.36X10^{-01}$	$5.87X10^{-24}$	Anaerobe
**Syntrophococcus**	2.240	2.212	$6.36X10^{-01}$	$1.40X10^{-24}$	Anaerobe
**Beijerinckia**	0.333	0.300	$6.46X10^{-01}$	$4.01X10^{-04}$	Anaerobe
**Holdemania**	1.450	1.494	$6.68X10^{-01}$	$5.70X10^{-07}$	Anaerobe
**Coprococcus**	2.787	2.810	$6.72X10^{-01}$	$2.41X10^{-25}$	Anaerobe
**Odoribacter**	2.184	2.162	$7.21X10^{-01}$	$2.72X10^{-29}$	Anaerobe
**Oscillibacter**	3.277	3.294	$7.21X10^{-01}$	$3.88X10^{-16}$	Anaerobe
**Anaerofilum**	0.852	0.821	$7.53X10^{-01}$	$2.51X10^{-07}$	Anaerobe
**Microbacterium**	0.285	0.311	$7.53X10^{-01}$	$6.54X10^{-18}$	Aerobe
**Howardella**	0.747	0.774	$7.65X10^{-01}$	$2.60X10^{-45}$	Anaerobe
**Hespellia**	0.266	0.279	$8.13X10^{-01}$	$4.58X10^{-05}$	Anaerobe

Bacteria (Genus level)	Sample Type			Participant	Culture Medium
	Stool Mean	Swab Mean	Adj. p-value	Adj. p-value	
**Solobacterium**	0.494	0.511	$8.32X10^{-01}$	$2.26X10^{-11}$	Anaerobe
**Lachnobacterium**	0.283	0.290	$8.99X10^{-01}$	$9.62X10^{-02}$	Anaerobe
**Eggerthella**	1.600	1.603	$9.06X10^{-01}$	$9.34X10^{-13}$	Anaerobe
**Barnesiella**	1.771	1.750	$9.43X10^{-01}$	$5.69X10^{-45}$	Anaerobe
**Aquabacterium**	0.689	0.694	$9.81X10^{-01}$	$2.10X10^{-43}$	Aerobe
**Flavonifractor**	2.667	2.668	$9.87X10^{-01}$	$2.54X10^{-05}$	Anaerobe
**Butyricimonas**	1.488	1.487	$9.90X10^{-01}$	$6.76X10^{-36}$	Anaerobe

Supplemental Table S5. Differences in WGS sequences due to sample type for bacterial species associated with colorectal cancer

Species	Mean			Sample Type Adjusted p-value	Participant Adjusted p-value
	Stool	Swab	Tissue		
Akkermansia muciniphila	1.86	1.61	0.63	$9.41x10^{-04}$	$1.50x10^{-10}$
Bacteroides fragilis	3.20	3.02	0.85	$1.45x10^{-33}$	$9.43x10^{-08}$
Bacteroides vulgatus	3.83	3.62	1.74	$5.93x10^{-24}$	$1.40x10^{-06}$
Bifidobacterium longum	1.47	1.24	0.11	$1.60x10^{-09}$	$6.26x10^{-07}$
Enterococcus faecalis	0.62	0.55	0.00	$3.02x10^{-07}$	$6.93x10^{-04}$
Escherichia coli	1.64	2.68	1.56	$1.26x10^{-05}$	$1.61x10^{-09}$
Eubacterium rectale	2.78	2.62	0.69	$5.92x10^{-18}$	$6.62x10^{-07}$
Faecalibacterium prausnitzii	3.13	3.18	1.18	$5.13x10^{-20}$	$6.77x10^{-04}$
Fusobacterium nucleatum	0.14	0.32	0.04	0.02	$6.77x10^{-04}$
Streptococcus thermophilus	1.05	0.81	0.00	$1.61x10^{-06}$	$3.96x10^{-04}$

Supplemental Table S6. Differences in WGS sequences due to sample type (stool vs. swab vs. mucosa) and participant source.

KEGG Pathway (Level 1)	Sample Type				Participant
	Stool Mean	Swab Mean	Mucosa Mean	adj p-value	Adj p-value
**Cellular Processes**	4.421	4.688	5.054	$1.18X10^{-29}$	0.999927
**Human Diseases**	3.468	3.807	4.851	$1.54X10^{-36}$	0.999927
**Organismal Systems**	3.561	4.220	5.364	$6.69X10^{-39}$	0.999927
**Genetic Information Processing**	5.265	5.251	5.119	$1.61X10^{-19}$	0.006560
**Environmental Information Processing**	4.452	4.701	4.721	$1.52X10^{-17}$	0.999927
**Metabolism**	5.997	5.943	5.792	$1.18X10^{-29}$	0.999927

Supplemental Table S7. Differences in WGS sequences due to sample type (stool vs. swab vs. mucosa) and participant source.

KEGG Pathway (Level 2)	Sample Type				Participant
	Stool Mean	Swab Mean	Mucosa Mean	p-value	Adj p-value
**Transcription**	3.915	3.907	3.569	$3.41X10^{-34}$	0.007886
**Translation**	4.720	4.713	4.923	$1.25X10^{-19}$	0.049822
**Glycan Biosynthesis and Metabolism**	4.823	4.793	4.625	$5.18X10^{-19}$	0.056813
**Immune System**	2.080	3.706	5.057	$3.15X10^{-38}$	0.060630
**Metabolism of Cofactors and Vitamins**	5.297	5.283	5.495	$1.51X10^{-26}$	0.291549
**Sensory System**	1.107	2.380	3.408	$8.99X10^{-31}$	0.291549
**Metabolism of Terpenoids and Polyketides**	4.510	4.432	3.016	$4.97X10^{-55}$	0.304266
**Signaling Molecules and Interaction**	2.370	3.964	4.445	$2.66X10^{-29}$	0.337843
**Environmental Adaptation**	3.097	3.335	4.043	$5.60X10^{-39}$	0.337843
**Cardiovascular Diseases**	0.474	2.194	3.772	$6.72X10^{-32}$	0.359203
**Nucleotide Metabolism**	4.478	4.406	3.920	$7.47X10^{-54}$	0.452661
**Cell Motility**	3.925	4.186	4.326	$4.03X10^{-17}$	0.452661
**Immune System Diseases**	0.716	1.985	4.023	$9.63X10^{-27}$	0.503557
**Digestive System**	3.116	3.544	4.741	$4.40X10^{-40}$	0.552044
**Lipid Metabolism**	4.711	4.691	4.519	$5.37X10^{-25}$	0.757339
**Carbohydrate Metabolism**	5.308	5.212	4.450	$2.28X10^{-50}$	0.757339
**Replication and Repair**	4.907	4.832	4.151	$4.91X10^{-51}$	0.877153
**Xenobiotics Biodegradation and Metabolism**	4.506	4.445	3.343	$2.59X10^{-19}$	0.999935
**Membrane Transport**	4.290	4.253	4.010	$1.33X10^{-12}$	0.999935
**Cell Growth and Death**	4.195	4.195	4.367	$3.32X10^{-18}$	0.999935
**Folding Sorting and Degradation**	4.623	4.670	4.437	$3.85X10^{-21}$	0.999935
**Cancers**	1.011	1.767	3.456	$7.04X10^{-31}$	0.999935
**Circulatory System**	0.490	1.968	3.402	$9.15X10^{-20}$	0.999935
**Transport and Catabolism**	2.918	3.642	3.921	$2.05X10^{-24}$	0.999935
**Development**	1.160	2.188	3.535	$1.12X10^{-28}$	0.999935
**Excretory System**	1.526	2.345	4.017	$5.35X10^{-22}$	0.999935
**Neurodegenerative Diseases**	2.202	2.929	4.431	$2.06X10^{-38}$	0.999935
**Metabolism of Other Amino Acids**	5.051	5.013	4.759	$2.30X10^{-10}$	0.999935

KEGG Pathway (Level 2)	Sample Type				Participant
	Stool Mean	Swab Mean	Mucosa Mean	p-value	Adj p-value
**Nervous System**	0.914	1.552	3.061	$9.91X10^{-14}$	0.999935
**Infectious Diseases**	3.415	3.549	3.545	$6.70X10^{-07}$	0.999935
**Signal Transduction**	3.849	4.020	4.120	$8.77X10^{-13}$	0.999935
**Biosynthesis of Other Secondary Metabolites**	4.406	4.285	1.896	$3.15X10^{-36}$	0.999935
**Amino Acid Metabolism**	5.266	5.189	4.984	$6.41X10^{-34}$	0.999935
**Endocrine System**	2.620	2.964	3.894	$2.32X10^{-22}$	0.999935
**Cell Communication**	0.770	3.280	4.764	$1.88X10^{-33}$	0.999935
**Energy Metabolism**	4.707	4.590	4.074	$1.21X10^{-39}$	0.999935
**Metabolic Diseases**	0.326	1.530	3.019	$6.56X10^{-26}$	0.999935
